# Crohn’s disease-associated AIEC inhibiting intestinal epithelial cell-derived exosomal let-7b expression regulates macrophage polarization to exacerbate intestinal fibrosis

**DOI:** 10.1080/19490976.2023.2193115

**Published:** 2023-03-21

**Authors:** Yihan Xu, Wenwei Qian, Liangyu Huang, Weiwei Wen, Yi Li, Feilong Guo, Zhenxing Zhu, Zhun Li, Jianfeng Gong, Zeqian Yu, Yan Zhou, Nan Lu, Weiming Zhu, Zhen Guo

**Affiliations:** a Department of General Surgery, Affiliated Jinling Hospital, Medical School of Nanjing University, Nanjing, China; b Department of General Surgery, Jinling Hospital, Medical School of Southeast University, Nanjing, China; c Department of Colorectal Surgery, The First Affiliated Hospital, Zhejiang University School of Medicine, Zhejiang, China

**Keywords:** Crohn’s disease, adherent-invasive*Escherichia coli*, macrophage, miRNA, intestinal fibrosis

## Abstract

The interaction between adherent-invasive *Escherichia coli* (AIEC) and intestinal macrophages is implicated in the pathogenesis of Crohn’s disease (CD). However, its role in intestinal fibrogenesis and the underlying molecular mechanisms are poorly understood. In addition, miRNAs such as let-7b may participate in AIEC-macrophage interactions. In this study, we identified that the colonization of AIEC in the ileum was associated with enhanced intestinal fibrosis and reduced let-7b expression by enrolling a prospective cohort of CD patients undergoing ileocolectomy. Besides, AIEC-infected IL-10^−/−^ mice presented more severe intestinal fibrosis and could be improved by exogenous let-7b. Mechanistically, intestinal macrophages were found to be the main target of let-7b. Transferring let-7b-overexpressing macrophages to AIEC-infected IL-10^−/−^ mice significantly alleviated intestinal fibrosis. In vitro, AIEC suppressed exosomal let-7b derived from intestinal epithelial cells (IECs), instead of the direct inhibition of let-7b in macrophages, to promote macrophages to a fibrotic phenotype. Finally, TGFβR1 was identified as one target of let-7b that regulates macrophage polarization. Overall, the results of our work indicate that AIEC is associated with enhanced intestinal fibrosis in CD. AIEC could inhibit exosomal let-7b from IECs to promote intestinal macrophages to a fibrotic phenotype and then contributed to fibrogenesis. Thus, anti-AIEC or let-7b therapy may serve as novel therapeutic approaches to ameliorate intestinal fibrosis.

## Plain Language Summary

What is the context?

Adherent-invasive *Escherichia coli* (AIEC) plays an important role in the pathogenesis of Crohn’s disease (CD) and has a strong association with intestinal fibrosis in animal models. However, how these bacteria contribute to intestinal fibrosis in CD patients is still unclear.

The plasticity of macrophages is crucial in immune tolerance and tissue repair in the gastrointestinal tract, and the abnormal interaction between macrophages and gut bacteria triggers the fibrogenesis in the intestine.

The association between the miRNA let-7b and fibrosis process has been widely reported in many tissues, except the intestine.

What is new?

AIEC colonization in the terminal ileum is associated with severe intestinal fibrosis in CD patients and the let-7b plays an anti-fibrotic role in intestinal fibrosis.

Intestinal macrophages are the key modulator of AIEC-induced fibrosis and can be promoted to an antifibrotic phenotype through let-7b-targeted TGFβR1 inhibition.

AIEC suppresses intestinal epithelial cell-derived exosomal let-7b to promote intestinal macrophages to a fibrotic phenotype, rather than a direct effect on macrophage regulation.

What is the impact?

- Anti-AIEC and let-7b therapy may serve as potential therapeutic strategies to reduce intestinal fibrosis in CD in the future.

## Introduction

More than half of patients with Crohn’s disease (CD) develop intestinal fibrosis during their lifetime, and subsequent stenosis and obstruction are common^1^. Although established biologics effectively reduce intestinal inflammation, no available antifibrotic drugs for CD currently exist. Thus, surgery remains the main therapeutic intervention for CD-associated intestinal fibrotic strictures^2^. Understanding the mechanisms of intestinal fibrosis in patients with CD is crucial to develop specific anti-fibrotic therapies.

The interplay between the gut microbiota and mucosal immunity contributes to the etiology of CD-associated intestinal fibrosis^3,4^. Although no single causative microorganism has been identified, some strains have been shown to play an important role in the pathogenesis of CD^5–7^. Specifically, a subset of resident intestinal *Escherichia coli*, known as CD-associated adherent-invasive *E. coli* (AIEC), is enriched in the terminal ileum of patients with CD and has a strong association with disease inflammatory activity and postoperative recurrence^8,9^. Emerging evidence has demonstrated that the colonization of AIEC results in both acute and chronic intestinal inflammation under favorable genetic or environmental conditions in animal models^8^. However, the association between AIEC and intestinal fibrosis in patients with CD remains unclear. Several studies have reported that AIEC-infected animals develop intestinal fibrosis^5,7,10^, but the molecular mechanisms have yet to be identified.

Macrophage (Mø) is one kind of the main target cell of AIEC and is a key modulator of fibrogenesis^11^. The critical role of Mø in fibrosis of the liver^12^, kidney^13^, and lung^14^ has consistently been demonstrated; however, the contribution of Mø to intestinal fibrosis is poorly understood. Hopefully, the hypothesis that the loss of tolerance to the gut bacteria evokes an excessive immune response in intestinal Mø and then initiates fibrogenic processes is being supported by accumulating evidence^11,15^. One study focusing on CD indicated that the accumulation of CD16+ Møs at the site of strictured intestine may be associated with enhanced intestinal fibrosis^16^. A previous study also found that AIEC could modulate Mø polarization to drive intestinal inflammation^17^. However, the impact of AIEC-Mø crosstalk on intestinal fibrosis and the underlying mechanism are not fully understood.

MicroRNAs (miRNAs) are noncoding single-stranded RNAs that can bind to complementary sequences in the 3’UTR of target mRNAs and regulate various physiological processes, including the gut microbiota-host interplay^18^. It has been demonstrated that AIEC can modulate microRNAs to regulate the intestinal mucosal immune response^19^. As shown in our previous study, the expression of the miRNA let-7b is suppressed in the terminal ileum of patients with active CD and restored to normal levels after inducing remission^20^. This result was confirmed by a recent study^21^. Furthermore, we found that the expression of let-7b in intestinal epithelial cells (IECs) was inhibited by AIEC infection, and exogenous let-7b ameliorated intestinal inflammation^22^. In addition, previous studies indicated that let-7b could modulate the polarization of Mø^23^ and played a role in kidney fibrosis^24^, suggesting the implication of let-7b in the AIEC-associated intestinal fibrosis.

In this study, we investigated the mechanism by which AIEC stimulated Mø polarization to a profibrotic characteristic through the inhibition of let-7b in the intestine, thereby inducing intestinal fibrosis.

## Results

### Intestinal fibrosis of the terminal ileum is severe in CD patients with AIEC colonization

Terminal ileum specimens were collected from 21 consecutive patients who underwent ileocolonic resection due to CD. The baseline characteristics of these patients are presented in Table 1. Among the 21 patients, 38 Enterobacteriaceae strains were isolated, and 18 strains from eight patients were identified as AIEC strains (Table S1). The prevalence of AIEC in this cohort was 38.1% (8/21).

**Table 1.** Demographic characteristics of patients in the cohort.

Characteristics	CD (*n* = 21)		
	AIEC (+) (*n* = 8)	AIEC (-) (*n* = 13)	P value
Age, yrs,	33.2 ± 13.1	35.6 ± 11.9	0.676
Gender, female, n (%)	4 (50.0%)	3 (23.1%)	0.204
Smoker, n (%)	2 (25.0%)	3 (23.1%)	0.920
HBI	3.8 ± 1.5	4.0 ± 1.8	0.874
Disease duration (month)	29.6 ± 28.6	35.6 ± 24.4	0.611
Montreal classification			
Age at diagnosis, n (%)			0.854
A1: < 17 yrs	0	0	
A2: 17–40 yrs	7 (87.5%)	11 (84.6%)	
A3: > 40 yrs	1 (12.5%)	2 (15.4%)	
Disease location, n (%)			0.466
L1: ileum	3 (37.5%)	7 (53.8%)	
L2: colon	0	0	
L3: ileocolon	5 (62.5%)	6 (46.2%)	
L4: upper gastrointestinal tract	0	0	
Disease behavior, n (%)			0.378
B1: inflammatory	0	0	
B2: stricturing	4 (50.0%)	9 (69.2%)	
B3: penetrating	4 (50.0%)	4 (30.8%)	
Perianal disease, n (%)	1 (12.5%)	2 (15.3%)	0.854
Medication, n (%)			0.408
5-Aminosalicylic acid	2	7	
immunosuppressants	1	0	
steroids	2	2	
biologics	3	4	
Antibiotics	0	0	1.000
Extraintestinal manifestations, n (%)	0	0	1.000

HBI, Harvey-Bradshaw Index. The quantitative data were presented as mean ± SEM. Fisher’s exact test was used for comparison between categorical data. Unpaired t test was used for comparison between numerical data. *P* values<0.05 were considered statistically significant.

**Figure 1.**
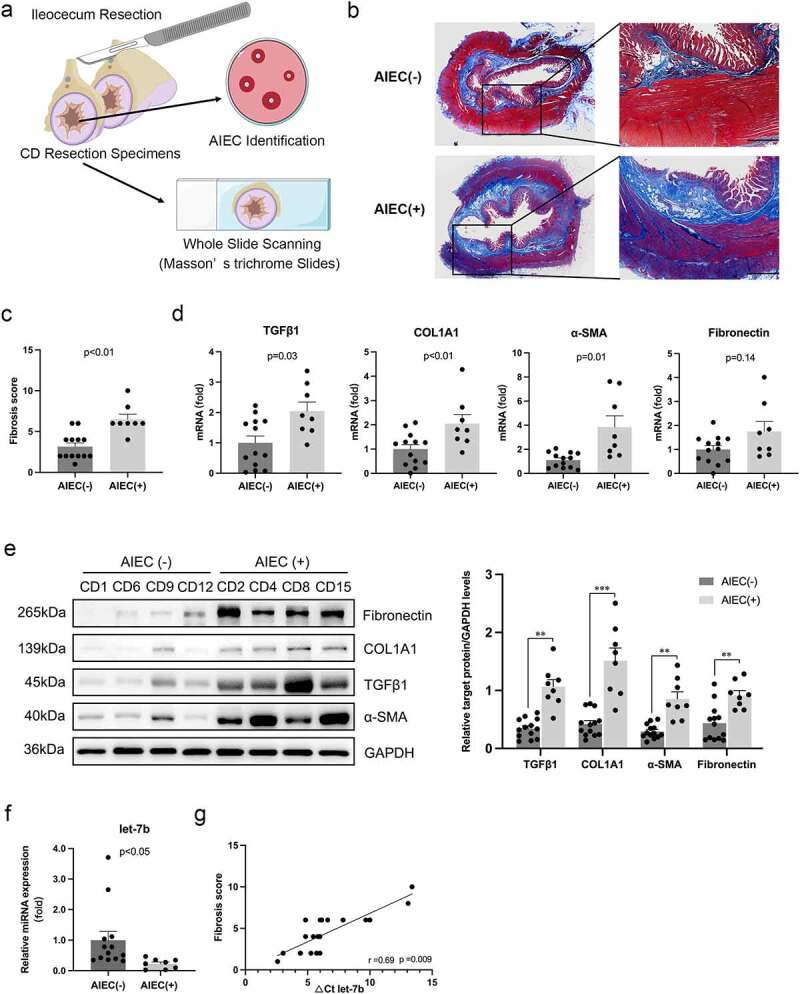
AIEC colonization is associated with the enhanced intestinal fibrosis in CD patients. (a) Illustration of the disposition of surgical resection specimens from CD patients. The slides of terminal ileum specimens are subjected to whole slide scanning to evaluate fibrosis after Masson’s trichrome staining, and mucosal samples are collected from the same sites of the specimens for AIEC identification. (b) Collagen fiber deposition in the submucosa and muscularis propria areas in AIEC(+) and AIEC(-) CD patients presented by Masson’s trichrome staining. Scale bar, 1 mm. (c) Fibrosis scores for quantification of degrees of intestinal fibrosis in AIEC(-) and AIEC(+) CD patients. (d&e) The mRNA and protein levels of TGFβ1, COL1A1, α-SMA and fibronectin in the terminal ileal tissues of AIEC(+) and AIEC(-) CD patients analyzed by qPCR and western blot, respectively. (f) The miRNA levels of let-7b analyzed by qPCR. U6 was used as an internal control. (g) Correlation between the levels of let-7b and fibrosis score in the terminal ileum. Spearman’s test was used for correlation analysis. AIEC(-): AIEC-negative colonization (*n* = 13); AIEC(+): AIEC-positive colonization (*n* = 8); data are expressed as the means ± SEM.

We performed Masson’s trichrome staining of whole terminal ileum sections to investigate the association between AIEC colonization and intestinal fibrosis (Figure 1a). Compared with AIEC-negative (AIEC(-)) patients, AIEC-positive (AIEC(+)) patients showed much more collagen deposition in the submucosal and muscularis propria layers of the terminal ileum (Figure 1b) and had a significantly higher fibrosis score (Figure 1c). In addition, both the mRNA and protein levels of the fibrotic markers, such as transforming growth factor β 1 (TGFβ1), alpha-smooth muscle actin (α-SMA) and collagen type I (COL1A1), were significantly increased in AIEC(+) patients with CD (Figure 1d–e).

Our previous study revealed that the miRNA let-7b level was decreased in the inflamed ileum of CD patients^20^. Here, we investigated whether severe intestinal fibrosis was associated with decreased expression of let-7b. Indeed, let-7b expression was clearly inhibited in AIEC(+) patients with CD (Figure 1f), and we observed a significant inverse correlation between let-7b expression and the fibrosis score of the terminal ileum (Figure 1g). These data suggested that AIEC colonization and decreased let-7b expression were associated with severe intestinal fibrosis in patients with CD.

### Persistent AIEC infection promotes intestinal fibrosis in IL-10^−/−^ mice

**Figure 2.**
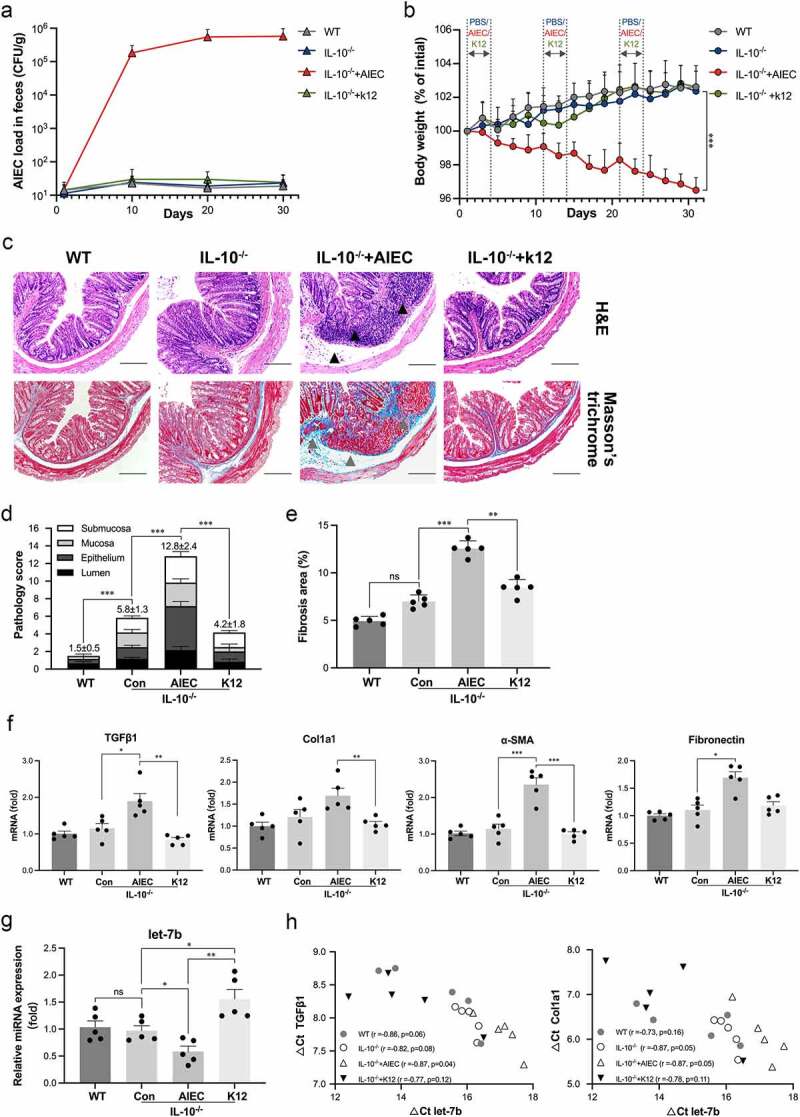
AIEC infection induced intestinal fibrosis and inhibited let-7b expression. (a) Quantification of AIEC strain LF82 (CFU/g) cultured from feces of WT and IL-10^−/−^ mice with AIEC/K12 strain infection at day 10, day 20, day 30. (b) Body weight of the mice measured every two days and expressed as a percentage of the initial weight. (c) H&E and Masson’s trichrome staining of colon sections from AIEC/K12-infected IL-10^−/−^mice and from uninfected controls. Black triangles indicate submucosal edema and inflammatory cellular infiltrates. Grey triangles indicate collagen deposition in the colonic submucosa and mucosa. Scale bar, 200 μm. (d) Colon pathology scored expressed as an average of five views per section. (e) Quantification of intestinal fibrosis expressed as the percentage of collagen deposition in colon samples using ImageJ software. (f) The mRNA levels of TGFβ1, Col1a1, α-SMA and fibronectin in the colon determined by qPCR. Data were normalized to mouse 36B4 RNA. (g) The miRNA levels of let-7b analyzed by qPCR. (h) Correlation between the levels of let-7b and degrees of intestinal fibrosis represented by mRNA levels of TGFβ1 and Col1a1. Spearman’s test was used for correlation analysis. Data are expressed as the means ± SEM, *n* = 5 in each group. *P<.05, **P<.01, ***P<.001.

To explore the mechanism of AIEC contributing to intestinal fibrosis, we developed a long-term and recurrent AIEC infection model using IL-10^−/−^ mice to mimic the chronic intestinal inflammation of patients with CD. Our results verified successful AIEC colonization in the mouse gut, which persisted throughout the whole experimental period (Figure 2a). In addition, IL-10^−/−^ mice infected with AIEC showed a significant decrease in body weight compared to control mice (WT and IL-10^−/−^ mice treated with PBS or nonpathogenic strain K12) (Figure 2b), as well as more severe macroscopic colon inflammation (Figure S1A).

The histological analysis confirmed that persistent AIEC colonization aggravated intestinal inflammation and fibrosis. Hematoxylin and eosin (H&E)-stained tissue sections showed that AIEC-infected colon tissue displayed CD-like transmural inflammation, severe inflammatory cellular infiltrates and submucosal edema (Figure 2c,d; Figure S1B). In addition, Masson’s trichrome staining revealed that AIEC infection induced massive collagen deposition compared to that in control mice (Figure 2c–e). Collagen deposition was mainly observed in the submucosal layer, resembling the characteristics of patients with CD with fibrotic stricture. Next, we evaluated fibrosis-related markers in colonic tissue to further characterize fibrosis induced by AIEC infection. Significantly increased expression of fibrotic markers, including TGFβ1, Col1a1, α-SMA and fibronectin, were observed in AIEC-infected animals at both transcriptional levels and protein levels (Figure 2f; Figure S1C)

To determine whether AIEC-induced fibrosis could affect the expression of let-7b in the intestine, we measured the levels of let-7b in the colon tissue. Similar to the results from patients with CD, let-7b expression was markedly decreased in AIEC-infected IL-10^−/−^ mice. Interestingly, infection with the K12 strain significantly increased the expression of let-7b (Figure 2g). Furthermore, the expression of let-7b showed an inverse correlation with that of the fibrosis indicators (TGFβ1 and Col1a1), especially in AIEC-infected group (Figure 2h). Based on these results, AIEC aggravated intestinal inflammation and fibrosis in IL-10^−/−^ mice, and let-7b had a potential role in AIEC-associated intestinal fibrosis.

### Let-7b overexpression ameliorates intestinal fibrosis

**Figure 3.**
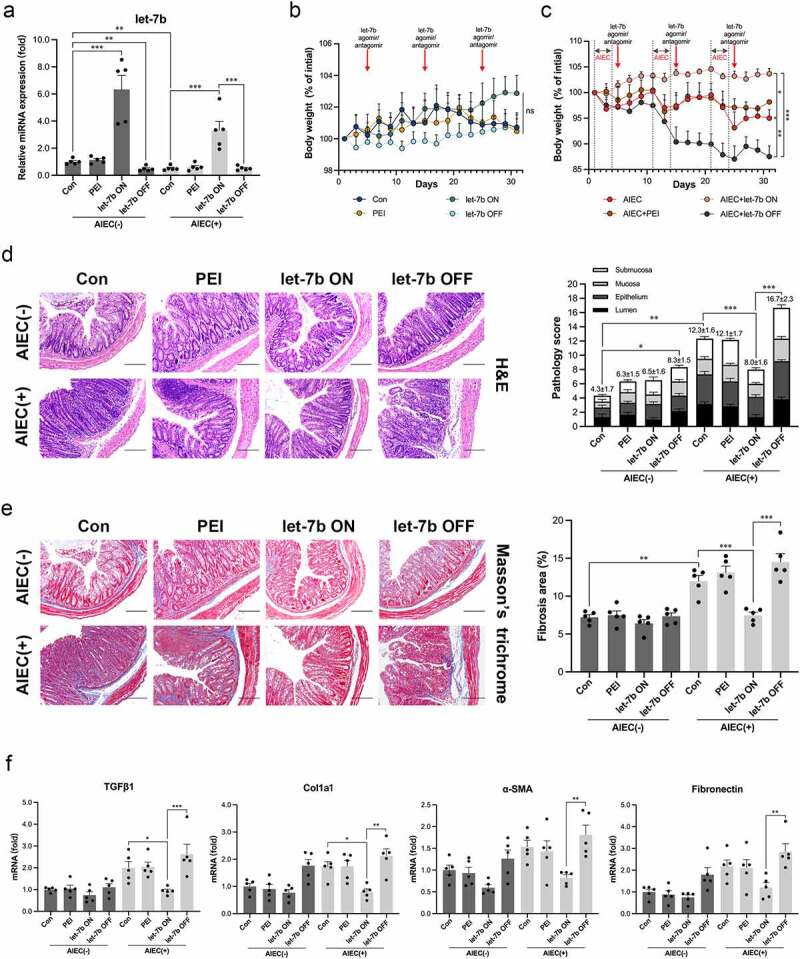
Upregulation of let-7b ameliorated intestinal fibrosis and inflammation. (a) The miRNA levels of let-7b in colon tissue in different groups measured by qPCR. (b&c) Body weight expressed as a percentage of initial weight. (d) H&E staining of colon sections and colon pathology scores and (e) Representative images and quantification of Masson’s trichrome staining of colon sections from IL-10^−/−^ mice after let-7b agomir/antagomir treatment with/without AIEC infection. Scale bar, 200 μm. (f) The mRNA levels of TGF-β1, Col1a1, α-SMA and fibronectin expression in the colon determined by qPCR. The data were normalized to mouse 36B4 RNA and expressed as fold-change relative to the IL-10^−/−^ mice control group. Let-7b ON: upregulation of let-7b with let-7b-agomir; let-7b OFF: inhibition of let-7b with let-7b-antagomir. Data are expressed as the means ± SEM, *n* = 5 in each group. *P<.05, **P<.01, ***P<.001.

To demonstrate whether let-7b exerts an antifibrotic effect on intestinal fibrogenesis, IL-10^−/−^ mice with/without AIEC infection were administered with let-7b agomir/antagomir dissolved in polyethyleneimine (PEI) to overexpress/silence let-7b expression in the intestine. As shown in Figure 3a, let-7b expression was decreased in AIEC-infected IL-10^−/−^ mice, and the let-7b agomir/antagomir successfully altered the levels of let-7b in AIEC-infected or noninfected IL-10^−/−^ mice. In addition, inhibition of let-7b with the let-7b antagomir (let-7b OFF) aggravated colitis in AIEC-infected mice, as reflected by obvious weight loss (Figure 3b,c), abnormal colon macroscopic manifestations (Figure S2A&B) and more severe histological changes and pathological scores (Figure 3d). In contrast, upregulation of let-7b with the let-7b-agomir (let-7b ON) alleviated colitis (Figure 3b-d). Next, we investigated whether let-7b ameliorated intestinal fibrosis development by staining tissues with Masson’s trichrome to visualize collagenous fiber deposition. As shown in Figure 3e, colon tissues from uninfected mice had a thin layer of collagen deposition restricted to the submucosal space, whereas AIEC infection caused extensive collagen deposition, especially in the let-7b OFF group. Conversely, animals in the let-7b ON group displayed minimal collagen deposition, which was confirmed by the quantification of the positive staining area. Additionally, we analyzed the expression of TGFβ1, Col1a1, α-SMA and fibronectin. The expression of these markers was strongly suppressed in the let-7b ON group, but was upregulated in the let-7b OFF group (Figure 3f; Figure S2C). Together, the obtained results suggested that let-7b was able to ameliorate intestinal fibrosis, particularly AIEC-induced intestinal fibrosis.

### Let-7b is enriched in IECs and Mø

**Figure 4.**
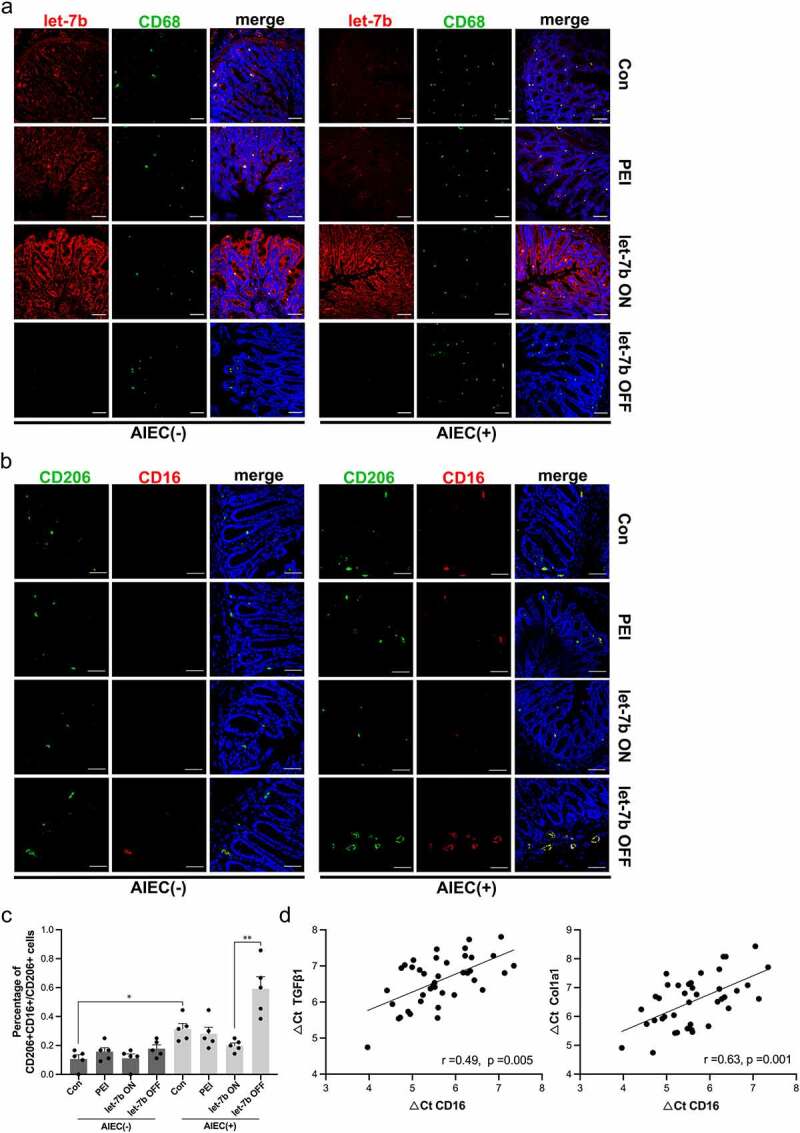
Localization of let-7b and Mø in colon tissue of IL-10^−/−^ mice. (a) Immunofluorescence staining for the Mø marker CD68 and FISH for let-7b were performed on AIEC-infected or noninfected IL-10^−/−^ mice treated with let-7b agomir/antagomir. (Red, let-7b; Green, CD68; Blue, DPAI). (b&c) Immunofluorescence analysis of the Mø phenotype. The Mø markers CD206 and CD16 were expressed on colon tissue from different groups. (Red, CD16; Green, CD206; Blue, DPAI). The ratio of profibrotic Mø was quantified by the number of CD16+CD206+ cells/CD206+ cells. (d) Correlation between the mRNA expression of CD16 and fibrotic markers TGFβ1 and Col1a1 in colon tissue. Spearman’s correlation analysis was applied. Data are expressed as the means ± SEM, *n* = 5 in each group. *P<.05, **P<.01.

To identify the potential target cells of let-7b in the intestine, fluorescence in situ hybridization (FISH) combined with immunofluorescence was performed. In colon tissues from noninfected IL-10^−/−^ mice, let-7b was mainly expressed in IECs at a moderate intensity, but its expression was inhibited in AIEC-infected colon tissue, indicating that AIEC suppressed the intestinal expression of let-7b (Figure 4a). In addition, after treatment with the let-7b agomir, abundant let-7b expression in the colon was strongly recovered, while let-7b expression was further intensively inhibited by the let-7b antagomir (Figure 4a). To investigate whether Mø is one of the targets of let-7b, we labeled intestinal Mø with anti-CD68 and found an accumulation of let-7b in Mø, suggesting that let-7b may have an effect on intestinal Mø (Figure 4a). The same phenomenon was also observed in the terminal ileum of CD patients (Figure S3A). Then we assessed the level of let-7b in Mø isolated from the lamina propria of the mouse intestine, and confirmed it was decreased in the AIEC-infected group (Figure S3B). In addition, *Salvador* and his colleagues reported that the number of CD206+CD16+ Mø was significantly increased in patients with CD presenting intestinal fibrosis^16^. Immunofluorescence staining of intestinal Mø was also performed to determine whether let-7b affected this subtype of Mø (Figure 4b,c). We found that AIEC infection increased CD16 expression on CD206+ Mø in the colon tissue. Notably, upregulating let-7b in AIEC-infected mice inhibited CD16 expression, and completely opposite results were observed in the let-7b OFF group (Figure 4b,c). Moreover, we confirmed the positive relationship between CD16 expression and fibrosis indicators (TGFβ1 and Col1a1) in the colon tissue (Figure 4d), and the association between CD16+CD206+ Mø and the intestinal fibrosis scores (Figure S3C). Therefore, intestinal Mø was one candidate target cell of let-7b, and let-7b inhibition caused by AIEC infection was associated with increased numbers of profibrotic Mø.

### Administration of let-7b-overexpressing peritoneal Mø to AIEC-infected mice alleviates intestinal fibrosis

**Figure 5.**
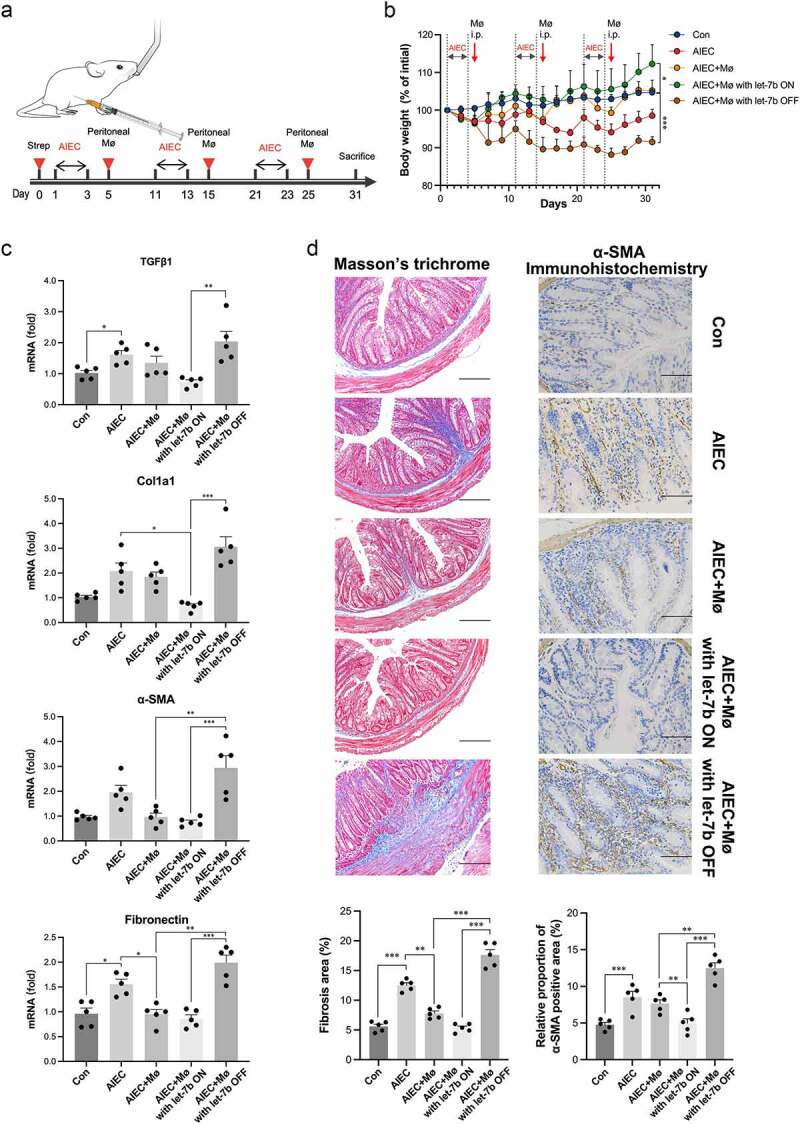
Transfer of let-7b agomir/antagomir pretreated Mø into AIEC-infected IL-10^−/−^ mice influenced intestinal fibrosis. (a) Illustration of transferring peritoneal Mø into AIEC-infected mice. (b) Body weight expressed as a percentage of initial weight. (c) The mRNA levels of TGFβ1, Col1a1, α-SMA and fibronectin in the colon were determined by qPCR. (d) Representative images and quantification of Masson’s trichrome staining (left panel) and representative images of immunohistochemical staining of α-SMA (right panel) from AIEC-infected IL-10^−/−^ mice transferred with peritoneal Mø pretreated with let-7b agomir/antagomir. Scale bar, 200 μm (left panel); 100 μm (right panel). Let-7b ON: upregulation of let-7b with let-7b-agomir; let-7b OFF: inhibition of let-7b with let-7b-antagomir. Data are expressed as the means ± SEM, *n* = 5 in each group. *P<.05, **P<.01, ***P<.001.

To identify whether Mø regulated by let-7b is a key modulator of intestinal fibrosis, peritoneal Møs obtained from IL-10^−/−^ mice were transfected with let-7b agomir/antagomir in vitro and then these Møs were intraperitoneally injected into IL-10^−/−^ mice 2 days after AIEC infection (Figure 5a). In previous studies, these retransferred Møs were shown to successfully migrate to the intestinal mucosa^16,25^. The injection of Møs transfected with the let-7b antagomir pretreatment caused significant weight loss in AIEC-infected mice and macroscopic colonic abnormalities. In contrast, the injection of let-7b agomir-pretreated Møs maintained weight and improved colitis (Figure 5b; Figure S4A). Moreover, AIEC-infected mice injected with let-7b agomir-pretreated peritoneal Møs showed decreased mRNA and protein expression of fibrotic indicators. Conversely, Møs transfected with the let-7b antagomir played a significant role in promoting intestinal fibrosis (Figure 5c; Figure S4B). These results were consistent with Masson’s trichrome staining reflecting collagen deposition and immunohistochemical staining for α-SMA reflecting fibroblast activation (Figure 5d). This part of the experiment confirmed that let-7b-targeted Møs were crucial for AIEC-induced intestinal fibrosis.

### Let-7b inhibits the profibrotic polarization of Mø

**Figure 6.**
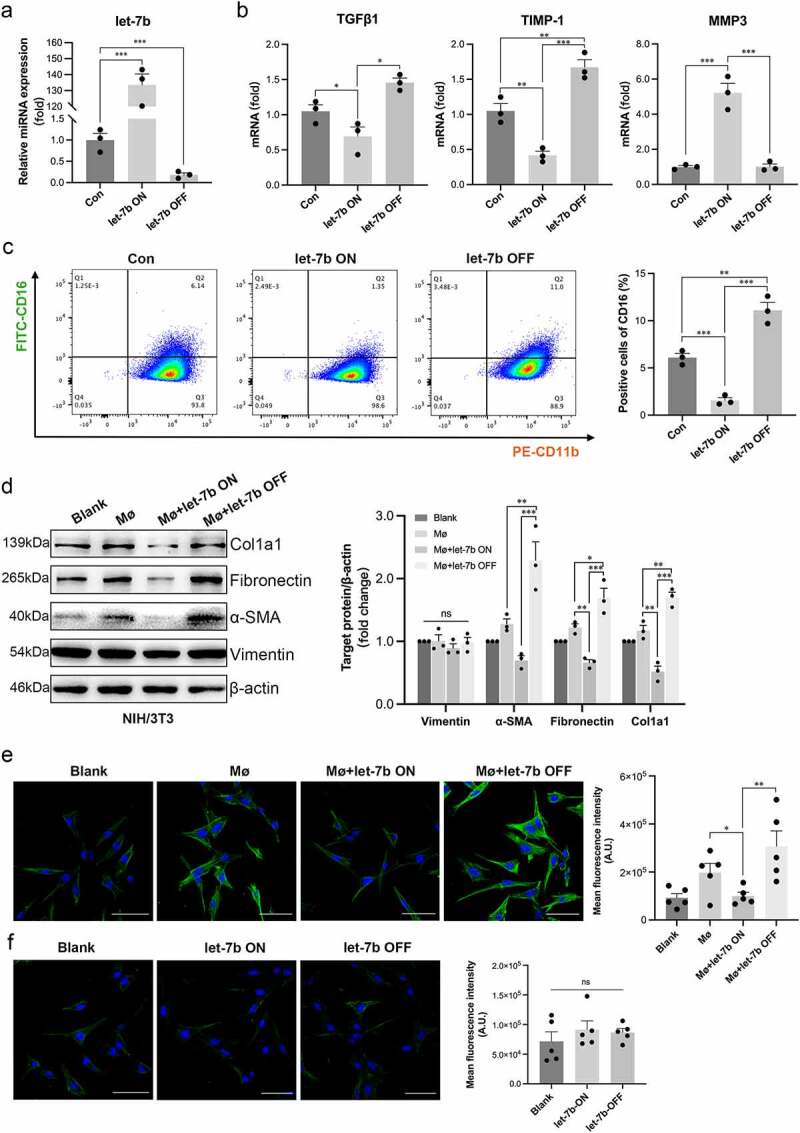
Let-7b inhibits the pro-fibrotic capacity of Mø. (a) The let-7b levels in RAW264.7 with different treatments determined by qPCR. (b) The mRNA levels of the pro-fibrotic markers TGFβ1 and TIMP-1 and the collagen degradation indicator marker MMP3 in RAW264.7 determined by qPCR. Data were normalized to mouse 36B4 RNA. (c) The percentage of CD16+CD11b+ Mø was analyzed by flow cytometry. (d) The expression levels of various proteins indicating the activation of fibroblasts in NIH/3T3 examined by western blot. The densitometric intensity of the bands was quantified using ImageJ software. (e) The expression of α-SMA in NIH/3T3 after coculture with RAW264.7 detected with immunofluorescence staining and quantified by fluorescence intensity with ImageJ software. Scale bar, 20 μm. (f) Immunofluorescence staining of α-SMA in let-7b agomir/antagomir-transfected NIH/3T3. Fluorescence intensity was quantified with ImageJ software. Scale bar, 20 μm. Let-7b ON: upregulation of let-7b with let-7b agomir; let-7b OFF: inhibition of let-7b with let-7b antagomir. Data are expressed as the means ± SEM. (a-d) All the experiments repeated 3 times, (e&f) all the experiments repeated 5 times. *P<.05, **P<.01, ***P<.001.

In vivo, we showed that let-7b exerted a direct effect on intestinal Mø to alleviate fibrosis. Thus, we conducted in vitro experiments to further investigate the regulatory effect of let-7b on Mø. We transfected RAW264.7 cells (a mouse peritoneal macrophage cell line) with the let-7b agomir/antagomir, and the efficiency of transfection was examined using quantitative real-time PCR (qPCR) (Figure 6a). As shown in Figure 6b, the mRNA levels of TGFβ1 and tissue inhibitor of metalloproteinases-1 (TIMP-1) in RAW264.7 were significantly suppressed after let-7b agomir treatment but were upregulated after let-7b antagomir transfection. In addition, the level of a marker of collagen degradation, matrix metalloproteinase-3 (MMP3), presented an opposite trend to TGFβ1 and TIMP-1. Consistently, we also observed the similar trend of TGFβ1, TIMP-1 and MMP3 expression at the protein levels (Figure S5A). Moreover, let-7b reduced the proportion of the CD16+ pool among RAW264.7 cells, according to the flow cytometry results (Figure 6c), consistent with the above mentioned in vivo results. Next, we established a coculture system consisting of RAW264.7 cells and NIH/3T3 fibroblasts to further confirm that Mø was the direct target of let-7b. The activation of NIH/3T3 cells was increased in the presence of RAW264.7 cells transfected with the let-7b antagomir, as reflected by western blotting and immunofluorescence staining (Figure 6d,e). However, the transfection of the let-7b agomir/antagomir into NIH/3T3 cells did not change the expression of the fibroblast activation marker α-SMA (Figure 6f). Taken together, let-7b exerted a direct effect on Mø polarization rather than on fibroblasts.

### AIEC regulates Mø polarization by inhibiting exosomal let-7b from IECs

**Figure 7.**
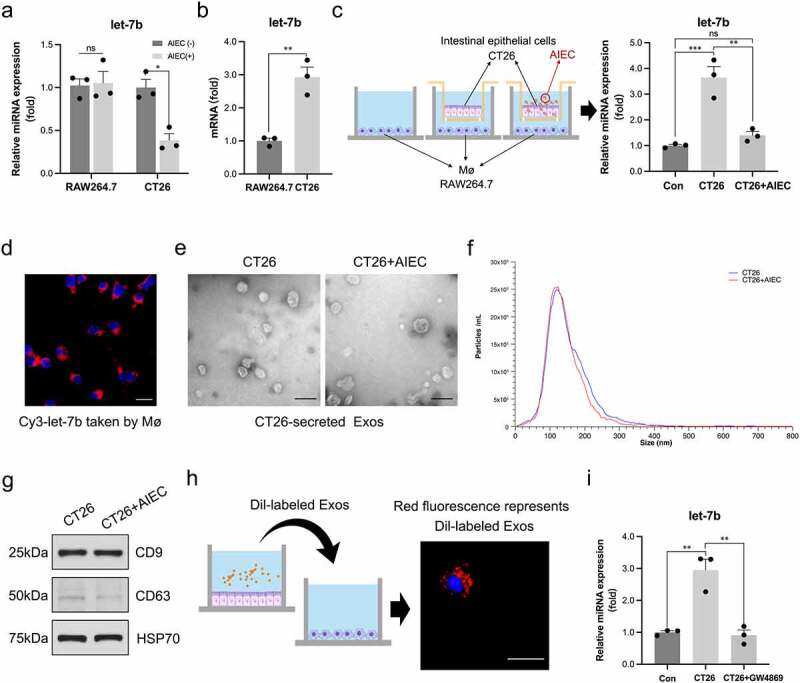
AIEC regulates Mø polarization by modulating IECs-derived exosomal let-7b. (a) The miRNA level of let-7b in RAW264.7 and CT26 with/without AIEC infection measured by qPCR. U6 was used as an internal control. (b) The original miRNA levels of let-7b in RAW264.7 and CT26. (c) The changes of let-7b levels in RAW264.7 after co-cultured with non-infected and AIEC-infected CT26. (d) The red fluorescence of the Cy3-labeled let-7b mimic transferred from CT26 to RAW264.7 detected by confocal microscopy. Scale bar, 10 μm. (e) Electron microscopy analysis of exosomes secreted by CT26. Scale bar, 100 nm. (f) Particle size of the vesicles secreted from CT26 measured by ZetaView analysis. (g) Exosome-associated protein markers HSP70, CD63, and CD9 measured by western blot analysis. (h) The red fluorescence of Dil-labeled CT26-derived exosomes detected by confocal microscopy. Scale bar, 50 μm. (i) The miRNA levels of let-7b in RAW264.7 co-cultured with CT26 pretreated with/without the exosome secretion inhibitor GW4689 (20 μM). Data are expressed as the means ± SEM. All the experiments repeated 3 times. *P<.05, **P<.01, ***P<.001.

We infected RAW264.7 cells with AIEC to explore whether AIEC directly affected Mø by reducing the production of let-7b. Interestingly, we did not observe a significant difference in let-7b expression in AIEC-infected RAW264.7 cells compared with the noninfected group (Figure 7a, left panel). However, our previous study confirmed that AIEC reduces let-7b expression in IECs^22^. Therefore, we hypothesized that AIEC modulates extracellular let-7b secreted by IECs to regulate intestinal Mø. To verify this, CT26 (a mouse colon epithelial cancer cell line) were exposed to the AIEC strain. The expression of let-7b was significantly decreased after AIEC infection (Figure 7a, right panel). Moreover, we observed that the expression of let-7b in RAW264.7 was much lower than that in CT26 (Figure 7b), and the abundance of let-7b increased 3-fold when RAW264.7 were co-cultured with CT26, but the let-7b expression was not noticeably changed when the CT26 were infected with AIEC in advance (Figure 7c). Additionally, we transfected CT26 cells with fluorescent Cy3-labeled let-7b mimics and then cocultured them with RAW264.7 cells to confirm that let-7b was transported from IECs to Mø (Figure 7d).

Exosomes are a major mechanism for miRNA transport. Thus, exosomes derived from noninfected and AIEC-infected CT26 cells were purified to verify whether exosomes acted as carriers of let-7b in IEC-Mø crosstalk. Electron microscopy (Figure 7e) and ZetaView analysis (Figure 7f) were applied and confirmed that the particles isolated by ultracentrifugation contained abundant CT26-derived exosomes with a diameter of 60–120 nm. Western blot analysis showed that the purified exosomes contained the exosomal markers CD9, CD63 and HSP70 (Figure 7g). We did not observe a difference in the micromorphology of CT26-derived exosomes after AIEC infection. Next, we tested whether CT26-derived exosomes were taken up by Mø. CT26-derived exosomes were labeled with the fluorescent dye Dil and then added to the culture medium of RAW264.7. After 6 h, RAW264.7 exhibited efficient uptake of CT26-derived exosomes, as detected by red fluorescence staining in these cells (Figure 7h). Furthermore, after co-culturing with CT26, a significant increase in let-7b expression was observed in RAW264.7. Importantly, when the exosome secretion inhibitor GW4869 was added to CT26 cells to block the exosome production, we observed a decrease of let-7b expression in RAW264.7, suggesting that CT26 secreted extracellular let-7b predominantly in an exosome-dependent manner (Figure 7i).

### Let-7b inhibits the profibrotic polarization of Mø by targeting TGFβR1

**Figure 8.**
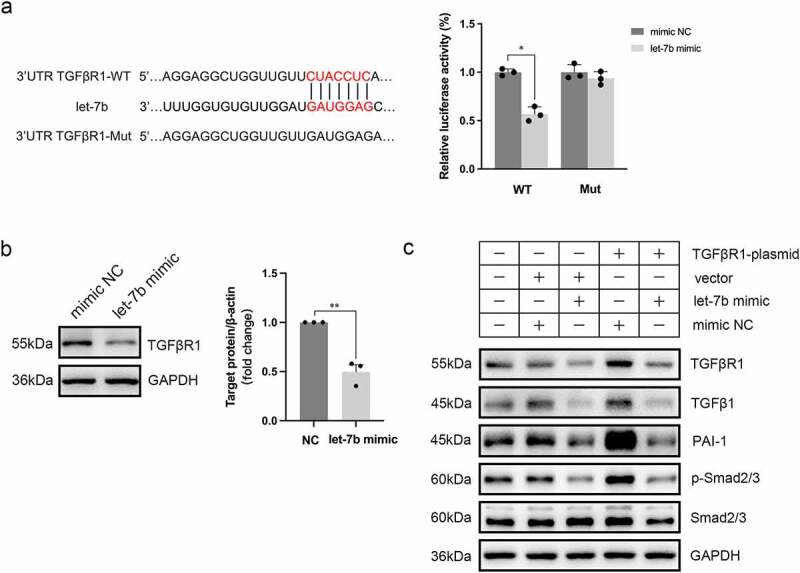
TGFβR1 is a direct target gene of let-7b. (a) Illustration of the complementary sequences between let-7b and TGFβR1 3’UTR and relative luciferase activity assayed by the ratio of firefly-renilla luciferase activity following transfection with let-7b mimic compared with transfection with mimic NC in 293T cells. (b) Protein expression of TGFβR1 in Møs after let-7b mimic transfection using western blot analysis. The densitometric intensity of the bands was quantified using ImageJ software. (c) Western blot analysis of the protein expression of TGFβR1, TGFβ1, PAI-1, *p*-Smad2/3, and Smad2/3 in Møs after different treatments. Data are expressed as the means ± SEM. All the experiments repeated 3 times. *P<.05, **P<.01.

We filtered let-7b binding sites in the 3’UTRs of transcripts encoding proteins involved in the fibrogenesis in Mø using online RNA databases (TargetScan, miRDB and miRBase), and TGFβ Receptor I (TGFβR1) was finally selected as a putative target of let-7b. We conducted dual-luciferase reporter gene assays to detect whether let-7b exerts a direct effect on TGFβR1 expression. The luciferase activity of TGFβR1-WT cells decreased upon let-7b mimic transfection, while the luciferase activity of TGFβR1-Mut cells did not change (Figure 8a). Additionally, western blot analysis also revealed a decrease in TGFβR1 protein levels in Mø upon let-7b mimic transfection (Figure 8b). These results indicated that let-7b targeted TGFBR1 and inhibited its expression. Inhibition of TGFβR1 has been reported to prevent or reduce fibrosis in many tissues^24,26,27^. To further demonstrate the role of let-7b in inhibiting fibrogenesis through the TGFβR1-dependent TGF-β/Smad signaling pathway in Mø, RAW264.7 were transfected with TGFβR1 plasmid and/or let-7b mimic. As shown in Figure 8c, TGFβR1 overexpression mediated the strong activation of the TGF-β/Smad signaling pathway, as reflected by increased levels of plasminogen activator inhibitor 1 (PAI-1) and phosphorylation of Smad2/3, while Smad2/3 phosphorylation and PAI-1 expression were effectively inhibited upon let-7b mimic transfection. In addition, the let-7b mimic also reduced TGFβ1 production in Mø contributing to the inhibition of TGF-β/Smad signaling pathway activation.

## Discussion

Here, we investigated the role of AIEC in intestinal fibrosis of CD. By enrolling a prospective cohort of patients with CD undergoing ileocolectomy, we observed much more severe intestinal fibrosis in the AIEC-infected terminal ileum, highlighting that ileal colonization by AIEC may be associated with enhanced CD fibrogenesis. In addition, AIEC colonization was also related to suppressed let-7b expression in the mucosa of the terminal ileum, and let-7b expression correlated inversely with the degree of intestinal fibrosis. Using in vivo and in vitro experiments, we confirmed that AIEC inhibited the secretion of exosomal let-7b from IECs, resulting in a reduction in the amount of let-7b delivered to Mø and the subsequent polarization of Mø to a profibrotic phenotype, thus exacerbating intestinal fibrosis. Finally, we identified that let-7b targeted TGFβR1, a key regulator of tissue fibrosis, to modulate the polarization of Mø.

AIEC survives and extensively replicates in Mø and then causes a significant alteration in cytokine production^28,29^. Compared with Mø from other organs, the phenotype of intestinal Mø is more complex^11^. Thus, intestinal Mø may not fit readily into the classical “M1/M2 paradigm”^30^. Additionally, previous studies have shown that levels of CD16+ circulating monocytes and tissue resident Mø may produce abundant TGFβ and are strongly associated with tissue fibrosis^31–33^. CD16+ Mø also exhibit higher phagocytic activity and express higher levels of proinflammatory cytokines upon bacterial infection^34,35^. *Salvador* and his colleagues further confirmed that this subgroup of Mø contributes to intestinal fibrosis by observing abnormal accumulation of CD16+ Mø in patients with CD complicated with fibrotic stenosis^16^. In our study, intestinal Mø were the key modulator of AIEC-associated intestinal fibrosis, and their effect on fibrosis was linked to CD16 expression, which was regulated by let-7b.

The let-7 family has been proven to participate in the interaction between bacteria and mucosal immunity^36^. In fact, let-7b also inhibits tissue fibrosis, such as renal fibrosis^24^, liver fibrosis^37^ and pulmonary fibrosis^38^, but the role of let-7b in intestinal fibrosis is unclear. In our previous study, let-7b expression was decreased in the mouse intestine infected with AIEC^22^. In this study, we strengthened this association in CD patients. Moreover, the let-7b levels were inversely correlated with the degree of intestinal fibrosis in patients with CD. We confirmed that exogenous let-7b reduced intestinal fibrosis in an AIEC-associated fibrosis model. In addition to the antifibrotic effect, the anti-inflammatory effect of let-7b was previously demonstrated^22,39^. As miRNA-based therapies are being investigated in clinical trials^40^, let-7b is a potential therapeutic strategy for intestinal inflammation and fibrosis in patients with CD.

Although let-7b is secreted by various cells^39^, we observed that let-7b was mainly enriched in IECs and Mø both in CD patients and IL-10^−/−^ mouse intestines. According to our results, AIEC infection did not directly affect the expression of let-7b in Mø. In contrast, we observed a significant decrease in let-7b expression in IECs after AIEC infection, consistent with our previous work^22^. These results indicated that AIEC may disrupt the crosstalk between IECs and Mø to induce fibrosis by modulating the secretion of let-7b by IECs. The interaction between IECs and Mø is one mechanism regulating the polarization of Mø^41^. As important secretory mediators, exosomes play a critical role in intercellular communication by delivering proteins and nucleic acids. *Jessica Carrière* et al. reported that the exosomes secreted by AIEC-infected IECs were intact, but the content was changed^41^. Our study confirmed these results. We also determined the regulatory role of let-7b by transferring IEC-derived exosomal let-7b to Mø, indicating that intestinal Mø require adequate IECs-secreted let-7b to maintain their immunoregulatory function under normal physiological conditions.

Finally, we evaluated the mechanism by which let-7b regulates the function of Mø. TGFβR1 is the key mediator modulating the TGF-β/Smad signaling pathway, which plays a major role in fibrotic processes in many tissues. In the Smad-dependent pathway, the interaction of TGFβ and TGFβR1 leads to downstream phosphorylation. It has been reported that let-7b targets the 3’UTR of TGFβR1 with a binding site to suppress fibrosis in the kidney^24^. Increased expression of let-7b inhibits Smad3 activity by upregulating TGFβRI expression^24^. In addition, *Tang* et al. also reported that hepatic fibrosis was distinctly ameliorated in a S. japonicum-infected mouse model, and TGFβ1 and TGFβR1 expression levels were markedly decreased after infection with a recombinant lentivirus expressing let-7b^37^. These findings support our results that let-7b interferes with the TGFβ/Smad signaling pathway in Mø by targeting TGFBR1.

Last but not least, several limitations existed in our study. First, only an AIEC-infected IL-10^−/−^ mouse model was used in this study, and we did not clearly determine whether let-7b reduced intestinal inflammation and fibrosis in other mouse models of colitis and intestinal fibrosis. We chose this model because it reflects the abnormal immune response to specific pathogens in individuals with genetic susceptibility, which adequately mimics the pathogenesis of CD. Second, both AIEC and let-7b affect various types of cells. We chose Mø because it is a key modulator of fibrogenesis and has been well studied in multiple organs, except the intestine. However, we cannot exclude the potential roles of other cells in AIEC-associated fibrosis. Last, one miRNA has more than one target mRNA, and TGFBR1 may not be the only gene regulated by let-7b in Mø to modulate its function.

In conclusion, AIEC is associated with enhanced intestinal fibrosis in patients with CD. AIEC aggravated intestinal fibrosis by promoting intestinal Mø polarization to a fibrotic phenotype by inhibiting IECs-secreted exosomal let-7b. Exogenous let-7b reduces AIEC-associated intestinal fibrosis. Therefore, anti-AIEC and let-7b therapies represent a potential strategy for CD.

## Methods

### Patients

From February 1st to July 31st, 2022, patients aged>17 years who were undergoing ileocolonic resection due to CD in Jinling Hospital were enrolled. The diagnosis of CD was based on endoscopy, radiology, and histology findings according to published criteria^42^. No patients received any antibiotic treatments within three months before surgery. Informed consent forms were signed by all participants, and the study protocol was approved by the Institutional Ethics Committee of Jinling Hospital (2016NZKY-013-02).

### Intestinal specimens

A section of approximately 1 cm of the intestinal tube was removed from the resected terminal ileum (3–5 cm next to the ileocecal valve) for histological analysis and AIEC identification. In detail, Masson’s trichrome staining was performed on whole terminal ileum sections to evaluate fibrosis. Meanwhile, multiple mucosal samples were collected from the same sites of the specimens for AIEC identification (Figure 1a).

### AIEC identification

Taking the previous work of *Darfeuille-Michaud* and other authors as references^43,44^. The identification of AIEC in this study met the following three conditions: i. the ability to adhere to IECs with an adhesion index (ADH_I) ≥ 1; ii. the ability to invade IECs with an invasion index (INV_I) ≥ 0.01; iii. the ability to survive and replicate within Mø with a replication index (REPL_I) > 100%. The details of the methods used to isolate and identify putative AIEC strains are provided in the supplementary materials.

### Establishment of the AIEC-infected mouse model

Eight- to ten-week-old wild-type (WT) and interleukin-10 knockout (IL-10^−/−^) male mice were obtained from the Model Animal Research Centre of Nanjing University (Nanjing, China). IL-10^−/−^ mice were then bred and maintained in pathogen-free cofferdam tanks for bacterial infection. First, IL-10^−/−^ mice were orally administered streptomycin (20 mg) for 3 days to disrupt the normal resident bacterial microbiota. Next, these mice were orally challenged with 200 μL of PBS containing 2 × 10^9^ colony-forming units (CFU) of AIEC reference strain LF82 or the nonpathogenic *E. coli* strain K12 MG1655 for 3 repeat cycles (3 days of bacterial infection followed by 7 days of normal feeding) using a previously described method, with some modifications^10^. WT and control animals received PBS. Animal protocols were approved by the Institutional Ethics Committee of Jinling Hospital (2021DZDWLS–007).

### Statistical analysis

The quantitative and qualitative variables were expressed as the mean ± SEM, frequency and percentage properly. All the statistical analyses were performed with Graphpad Prism 8.0 software (GraphPad Software Inc., USA). The unpaired Student’s t-test was used for comparison between two groups, and a standard one or two-way ANOVA was used for comparisons of three or more groups depending on the experimental design. Correlation analysis was done using a two-tailed Spearman correlation analysis. Differences with p-values<0.05 were considered statistically significant. All experiments were performed at least three times.

## Supplementary Material

Supplemental Material

## Data Availability

The datasets and methodology for screening miRNA targets are uploaded to the Figshare universal data repository (https://doi.org/10.6084/m9.figshare.22299679.v2). Additional information are available from the corresponding author upon request.
