# Supplementary material for: Crohn’s disease-associated AIEC inhibiting intestinal epithelial cell-derived exosomal let-7b expression regulates macrophage polarization to exacerbate intestinal fibrosis
Source: Gut Microbes. 2023 Mar 21;15(1):2193115. doi: 10.1080/19490976.2023.2193115 (PMC10038049; doi:10.1080/19490976.2023.2193115)
Supplement: Supplemental Material [file KGMI_A_2193115_SM6628.zip › 2.fully_revised_no_link_Methods_supply_Gutmicrobe.docx]

**Supplementary materials**

**Methods**

1. Specimen disposition and enteric *E. coli* isolation

Multiple mucosal biopsies collected from the same sites of specimens were immediately incubated with Roswell Park Memorial Institute (RPMI)-1640 medium containing 100 μg/ml gentamicin at 37 °C for 1 h to eliminate extracellular microbiota and then ultrasonically shocked three times with sterile saline to discard loosely attached bacteria. Next, the samples were lysed in 200 μl of 1% Triton-X-100/PBS for 8 min to release intracellular bacteria. The total samples and homogenate were plated on MacConkey agar differential medium and incubated at 37 °C for 18 h. A single colony forming unit of *E. coli* was selected and grown in Luria Bertani (LB) broth under aerobic conditions for 4 h at 37 °C.^1^ Amplified bacteria were stored at -80 °C in 50% (v/w) LB/glycerol for further AIEC identification.

2. AIEC identification

2.1 Adhesion and invasion assay

Caco-2 cells were seeded in 24-well plates at a density of 2×10^5^ cells/well and incubated for 24 h at 37 °C with 5% CO_2_ until reaching semi confluence (70-80%). Before bacterial infection, the cell culture medium was replaced with complete medium without antibiotics. Bacterial invasion of Caco-2 cells was measured using the gentamicin protection assay.^2^ For adhesion, cells were incubated for 3 h at 37 °C with 5% CO_2_, washed three times with PBS and then lysed with 1% Triton X-100/PBS in the absence of gentamicin protection. The suspension was diluted and plated onto MacConkey agar differential medium to determine the number of CFU. INV_I was calculated as the number of intracellular bacteria compared with the initial inoculum, which was defined as 100%. ADH_I was calculated as the number of bacteria per cell.^3^ Bacteria were considered able to adhere and invade intestinal epithelial cells when ADH_I ≥ 1 and INV_I ≥ 0.01.

2.2 Bacterial survival and replication within Mø

The survival and replication of bacteria in Mø were assessed using the gentamicin protection assay.^4^ Bacteria were considered able to survive and replicate in Mø when REPL_I > 100% (intracellular bacteria (t=24 h)/intracellular bacteria (t=1 h) ×100%).

3. Bacterial strains and cell culture

*E. coli* strain LF82, which was used as the reference strain for AIEC, has been widely used in AIEC research to date,^2, 5^ and our LF82 strain was kindly provided by Université Clermont Auvergne (Clermont-Ferrand, France). The commensal *E. coli* K12 strain MG1655 (ATCC) was used as a nonpathogenic reference. Bacterial strains were grown in LB broth on a shaker at 37 °C. During the log phase, bacteria were harvested and resuspended in phosphate-buffered saline (PBS) after centrifugation for 5 min at 6000 × g for the in vivo and in vitro bacterial infection experiments. Cell lines, including RAW264.7, NIH/3T3, CT26 and Caco-2 cells, were originally obtained from the National Collection of Authenticated Cell Cultures (Shanghai, China). RAW264.7 and NIH/3T3 cells were cultured in Dulbecco's modified Eagle’s medium (DMEM) (Gibco, USA). CT26 cells were cultured in RPMI-1640 medium (HyClone, USA). Caco-2 cells were maintained in minimum essential medium (MEM) (Gibco, USA) containing 2 mM L-glutamine, 100 mM sodium pyruvate, and 1% nonessential amino acids. All media were supplemented with 10% foetal bovine serum (FBS) and 1% penicillin/streptomycin. Cells were cultured at 37 °C in a 5% CO_2_ humidified incubator to 70-80% confluence.

4. MiRNA transfection in vivo

IL-10^-/-^ mice were treated with let-7b agomir/antagomir complexes as previously described.^6^ Briefly, 4 mg/ml polyethyleneimine (PEI) (25 kDa, Sigma, USA) solution and 2 mg/ml let-7b agomir/antagomir (Ribobio, China) in double distilled water (ddH_2_O) were mixed in an equal volume. According to our previous experience,^7^ PEI/let-7b agomir (5 mg/kg), PEI/let-7b antagomir (10 mg/kg), PEI alone or sterile PBS was administered to the colonic lumen of anaesthetized IL-10^-/-^ mice via a catheter inserted through the anus at the end of each AIEC infection cycle. The mice were then placed upside down for 1 min. Meanwhile, the IL-10^-/-^ mice without AIEC infection received the same interventions as the negative controls. The weight of the animals was evaluated at the beginning of the experiment and every 2 days thereafter. Colon tissues were collected for protein and RNA extraction or histological analyses.

5. Evaluation of bacterial colonization

Bacterial persistence in the gut was evaluated before any treatment (Day 0) and at the end of each bacterial infection cycle (Day 10, 20, and 30) to verify and quantify the colonization of bacteria in mice. Specifically, fresh faecal samples were collected (50-100 mg) and homogenized in the proper volume of PBS. The number of AIEC was determined by quantitative planting of serial dilutions of faeces on MacConkey agar differential medium containing 50 μg/ml ampicillin and 25 μg/ml erythromycin because AIEC strain LF82 is resistant to these antibiotics, and no resistant bacteria were isolated from uninfected mice.^8^ Plates were incubated overnight under aerobic conditions at 37 °C, and the number of bacterial colonies was enumerated by counting CFU.

6. Cell infection and transfection

Cells were seeded on culture plates without antibiotics one day before bacterial infection. Bacteria were grown in LB broth at 37 °C with shaking to the logarithmic phase. Bacterial infection was performed as previously described.^9^ Briefly, RAW264.7 and CT26 cells were inoculated with AIEC LF82 or *E. coli* K12 MG1655 at a multiplicity of infection (MOI) of 10. Three hours after the infection, the cells were washed three times with warm PBS and recultured in their respective medium with an additional 100 μg/ml gentamicin for another 24 h to eliminate extracellular bacteria. The let-7b agomir and antagomir (Ribobio, China) were transfected into recipient cells with riboFECT™ CP Transfection Kits (Ribobio, China) as described in our previous article.^7^ After 24 h, the transfection efficiencies were evaluated by performing a qPCR analysis. Total RNA and protein were extracted for qPCR and western blot analyses, respectively, and cell culture supernatants were collected for exosome extraction.

7. Isolation and retransfer of peritoneal Møs

Germfree IL-10^-/-^ mice were sacrificed by carbon dioxide inhalation, and 10 ml of precooled PBS was injected into the peritoneal cavity. The suspension was then withdrawn and centrifuged for 5 min at 400 g at 4 °C. The pellets were resuspended in RPMI-1640 medium supplemented with 1% penicillin‒streptomycin and 10% FBS, and cells were seeded in petri dishes and incubated for 4 h at 37 °C. Nonadherent cells were subsequently removed by washing with PBS, and the remaining adherent cells were Møs. Next, Møs were transfected with the let-7b agomir (50 nM/ml) and antagomir (100 nM/ml) (Ribobio, China) for another 48 h according to the manufacturer’s manual. They were then digested with trypsin-EDTA (Gibco) and washed twice with warm RPMI-1640 medium. Møs (2×10^6^) were injected intraperitoneally with a 19 G needle 2 days after each AIEC infection cycle.

8. Protein extraction and western blot analysis

The cells were lysed in RIPA buffer containing a protease inhibitor cocktail (Thermo, USA). The same amounts of proteins were loaded on 10% SDS‒PAGE gels to separate the target proteins. Proteins were then transferred onto PVDF membranes (Millipore, USA), blocked with 5% nonfat milk (Nestle, Switzerland) at room temperature and incubated with the primary antibodies overnight at 4 °C. The sources of primary antibodies were as follows: TGFβ1 (ab215715, Abcam), TGFβR1 (ab235578, Abcam), Smad2/3 (ab202445, Abcam), p-Smad2/3 (ab254407, Abcam), PAI-1 (ab222754, Abcam), Collagen Type I (14695-1-AP, Proteintech), α-SMA (ab32575, Abcam), fibronectin (15613-1-AP, Proteintech), Vimentin (60330-1-Ig, Proteintech), TIMP-1 (ab179580, Abcam), MMP3 (17873-1-AP, Proteintech), CD16 (16559-1-AP, Proteintech), CD206 (60143-1-Ig, Proteintech), CD68 (28058-1-AP, Proteintech), GAPDH (60004-1-Ig, Proteintech), and β-actin (A5441, Sigma). After 3 washes, the membranes were incubated with the appropriate HRP-conjugated secondary antibodies for 1 h at room temperature. The blots were detected using an Automated Chemiluminescence/Fluorescence Image Analysis System (Tanon, China).

9. Total RNA extraction and qPCR

Total RNA, including mRNAs and miRNAs, was extracted from colonic tissues and cells using TRIzol reagent (Takara, Japan). Reverse transcription of mRNAs to cDNAs was performed with HiScript III RT SuperMix for qPCR (Vazyme, China), while reverse transcription of miRNAs to cDNAs was conducted with a riboSCRIPT™ Reverse Transcription Kit (Ribobio, China). qPCR was performed using a LightCycler® 96 System with ChamQ SYBR qPCR Master Mix (Vazyme, China) to examine the target mRNA and let-7b levels according to the manufacturer’s manual. Relative gene expression was reported as fold induction: change in expression (fold) = 2^-ΔΔCT^, where ΔCT = CT (target)-CT (housekeeping), and ΔΔCT = ΔCT (treated)-ΔCT (control). The human GAPDH mRNA and mouse 36B4 or U6 RNA were used as housekeeping genes for normalization of target mRNA and let-7b expression. The primer sequences are listed in Table S3.

10. Histological analysis and immunohistochemical staining

Human and mouse intestinal tissues were maintained in 10% neutral buffered formalin for 48 h. They were then fixed, embedded in paraffin, sectioned (4 μm), mounted on glass slides and stained with H&E for the analysis of the structure and Masson’s trichrome to evaluate collagen deposits. The histopathological scores were calculated using previously defined criteria and descriptions.^10^ Intestinal fibrosis was defined as the mean proportion of collagen deposition in multiple sections from the same tissue and was calculated using ImageJ software (NIH, USA).^11^ Intestinal sections were stained with an antibody against α-SMA and then visualized with ImageJ software to examine the activation of myofibroblasts, which reflects the degree of intestinal fibrosis. α-SMA-positive cells were quantified as a percentage of the total area in the same field of view.^12^

11. Immunofluorescence staining

After fixation with 4% paraformaldehyde and permeabilization with 0.1% Triton X-100 (Sigma‒Aldrich) in PBS, NIH/3T3 cells and mouse colon tissue cells were incubated with specific primary antibodies overnight at 4 °C. The following primary antibodies were used at the recommended dilutions: α-SMA, CD206 and CD16. The samples were then stained with Alexa Fluor 488- and Alexa Fluor 594-labelled secondary antibodies (Sigma) for 60 min at room temperature. Hoechst 33342 (Thermo) was used as a nuclear dye and incubated for 10 min. Finally, the cells and tissues were photographed with a Zeiss LSM880 laser confocal microscope (Zeiss, Germany). For the analysis of the mean fluorescence intensity (MFI) of α-SMA, 5 different fields of each sample of NIH/3T3 cells were randomly selected and then the results were calculated using ImageJ software. The presence of profibrotic Mø subtypes is presented as the ratio of CD206+CD16+ cells/CD206+ Mø.^13^

12. Isolation of mouse lamina propria Mø

The cells isolation of colonic lamina propria were described previously.^14^ Briefly, we cut the colons into small pieces and then the colons were incubated in Ca/Mg-free EDTA/HBSS (Gibco, USA) at 37°C for 10 min twice in the 200 rpm shaker to remove IECs. The remaining tissues were then digested in 1 mg/ml Collagenase I (Roche, Germany) and and 0.05 mg/ml DNase (Roche, Germany) in RPMI 1640 medium for 30 min at 37°C. Next, the supernatants were filtered through 100 μm and then 40 μm cell strainers to obtain a single mononuclear cell suspension. The F4/80+ cells were further purified using immunomagnetic microbead technology (Miltenyi, Germany) to investigate the amount of let-7b in colonic macrophages, according to the manufacturer's instructions.^15^

13. Flow cytometry analysis

After transfection with the let-7b agomir/antagomir, RAW264.7 were washed twice with FACS buffer (HBSS containing 10% FBS, 5 mM EDTA and 20 mM HEPES) and then stained with a PE-conjugated anti-mouse CD11b antibody (Biolegend) and FITC-conjugated anti-mouse CD16 antibody (Biolegend) for 30 min at 4 °C in the dark. Stained RAW264.7 were resuspended in FACS buffer and then separated using a BD LSRFortessa Flow Cytometry Analyser (BD, USA). Data were analysed using FlowJo software (BD Biosciences).

14. FISH of miRNAs and immunofluorescence staining for Mø markers

The localization of miRNA let-7b in mouse intestinal tissues was detected using FISH, as previously described.^6^ Briefly, paraffin-embedded colon tissues were sectioned at 5 μm and deparaffinized. Prior to hybridization, prehybridization buffer (1× PBS/0.5% Triton X-100, Sigma) was added to each tissue section and incubated for 1 h at 37 °C. Hybridization was maintained overnight at 55 °C in a humidified chamber with hybridization buffer (formamide, 50 mM Tris-HCl, 5 mol NaCl, and 0.05% sodium dodecyl sulfate) containing a fluorescently labelled probe for let-7b (5′-Cy3-AACCACACAACCUACUACCUCA). Scrambled probes were used as controls. The same tissue sections were incubated with the anti-CD68 antibody overnight at 4 °C and then stained with Alexa Fluor 488-labelled secondary antibodies for 60 min at room temperature to localize the Mø in the intestinal tissue. Next, diluted DAPI (ab104139, Abcam) was added and incubated for 5 min to stain the nucleus. All images of fluorescence were captured using a Zeiss LSM880 laser confocal microscope.

15. Exosome isolation and characterization

Supernatants of RAW264.7 and CT26 were collected at the indicated time points after AIEC infection, and exosomes were extracted as previously described.^16^ Briefly, the cell culture supernatants were centrifuged at 300 × g for 10 min at 4 °C followed by 2,000 × g for 20 min to remove cell fragments. The remaining supernatants were centrifuged at 10,000 × g for 30 min to remove the subcellular components and then ultracentrifuged at 100,000 × g for 2.5 h to deposit the exosomes. The final exosomal pellet was resuspended in PBS. Transmission electron microscopy (Tecnai G2 Spirit Bio-Twin, FEI Co., USA) was applied to identify the appearance of exosomes. In addition, the levels of exosome-associated protein markers HSP70, CD63, and CD9 were confirmed by western blotting, and the exosome particle size was measured using a ZetaView Nanoparticle Tracking Analyser (Particle Metrix, Germany). Exosomes were tracked and detected by incubating them with the lipophilic membrane dye Dil (Invitrogen) for 1 h. After Dil staining, the exosomes were washed with PBS and collected by ultracentrifugation (100,000 × g for 20 min) at 4 °C before an incubation with recipient cells.

16. Cell co-culture and Transwell experiments

After infection with AIEC for 3 h, CT26 were seeded at a density of 5×10^5^ cells per well in the upper chamber of the Transwell plate (0.4 mm polycarbonate filter, Corning). Meanwhile, 5×10^5^ RAW264.7 were plated in the lower chamber and co-cultured with CT26 for 24 h. Next, RAW264.7 cells were harvested to examine the change in let-7b expression by performing qPCR analysis. CT26 alone and the blank upper layer without any treatment were used as the control groups. CT26 cells were transfected with Cy3-labelled let-7b mimic in the lower chamber and co-cultured with RAW264.7 cells for 24 h to further validate the source of let-7b. After two washes with PBS, the presence of red fluorescence of Cy3 in RAW264.7 cells was verified using a Zeiss LSM880 laser confocal microscope. GW4869 (a neutral sphingomyelinase inhibitor to restrict exosome biogenesis/release) (HY-19363, MedChemExpress) was added to CT26 cell culture medium to inhibit exosome secretion.^16^ These CT26 were collected and cocultured for another 12 h with RAW264.7 in medium containing GW4869.

17. Overexpression through plasmid transfection in vitro

The TGFβR1 and empty vector plasmids were purchased from GenePharma (Shanghai, China). RAW264.7 were seeded in 6-well plates (2×10^5^ cells/well) and incubated for 24 h to reach 60-70% confluence. Transient transfection of TGFβR1 and empty vector plasmids was performed with Lipofectamine™ LTX (15338100, Thermo) according to the manufacturer’s instructions. Briefly, 2.5 μg of plasmid DNA and 6.25 μl of transfection reagent were mixed and added to the cell medium without antibiotics. After 24 h of incubation, the cell medium was replaced with fresh complete culture medium to maintain cell growth for another 24 h before harvest.

18. Dual luciferase activity assay

The TGFβR1 3’UTR containing wild-type (WT) or mutant (Mut) let-7b binding sites was inserted into pGL3-luciferase reporter vectors (GenePharma, China). The pGL3-TGFβR1-WT or pGL3-TGFβR1-Mut plasmid along with the let-7b mimic or mimic NC were cotransfected into 293T cells using GP-transfect-Mate agent (GenePharma, China). Cells were harvested 48 h after transfection, and luciferase activity was assessed using the Dual-Luciferase Reporter Assay Kit (GenePharma, China) according to the manufacturer’s instructions.

**References:**

1. De la Fuente M, Franchi L, Araya D, Diaz-Jimenez D, Olivares M, Alvarez-Lobos M, et al. Escherichia coli isolates from inflammatory bowel diseases patients survive in macrophages and activate NLRP3 inflammasome. Int J Med Microbiol 2014; 304:384-92.

2. Darfeuille-Michaud A, Boudeau J, Bulois P, Neut C, Glasser AL, Barnich N, et al. High prevalence of adherent-invasive Escherichia coli associated with ileal mucosa in Crohn's disease. Gastroenterology 2004; 127:412-21.

3. Lopez-Siles M, Camprubi-Font C, Gomez Del Pulgar EM, Sabat Mir M, Busquets D, Sanz Y, et al. Prevalence, Abundance, and Virulence of Adherent-Invasive Escherichia coli in Ulcerative Colitis, Colorectal Cancer, and Coeliac Disease. Front Immunol 2022; 13:748839.

4. Glasser AL, Boudeau J, Barnich N, Perruchot MH, Colombel JF, Darfeuille-Michaud A. Adherent invasive Escherichia coli strains from patients with Crohn's disease survive and replicate within macrophages without inducing host cell death. Infect Immun 2001; 69:5529-37.

5. Darfeuille-Michaud A, Neut C, Barnich N, Lederman E, Di Martino P, Desreumaux P, et al. Presence of adherent Escherichia coli strains in ileal mucosa of patients with Crohn's disease. Gastroenterology 1998; 115:1405-13.

6. Shi T, Xie Y, Fu Y, Zhou Q, Ma Z, Ma J, et al. The signaling axis of microRNA-31/interleukin-25 regulates Th1/Th17-mediated inflammation response in colitis. Mucosal Immunol 2017; 10:983-95.

7. Guo Z, Cai X, Guo X, Xu Y, Gong J, Li Y, et al. Let-7b ameliorates Crohn's disease-associated adherent-invasive E coli induced intestinal inflammation via modulating Toll-Like Receptor 4 expression in intestinal epithelial cells. Biochem Pharmacol 2018; 156:196-203.

8. Chassaing B, Koren O, Carvalho FA, Ley RE, Gewirtz AT. AIEC pathobiont instigates chronic colitis in susceptible hosts by altering microbiota composition. Gut 2014; 63:1069-80.

9. Carriere J, Bretin A, Darfeuille-Michaud A, Barnich N, Nguyen HT. Exosomes Released from Cells Infected with Crohn's Disease-associated Adherent-Invasive Escherichia coli Activate Host Innate Immune Responses and Enhance Bacterial Intracellular Replication. Inflamm Bowel Dis 2016; 22:516-28.

10. Coburn B, Li Y, Owen D, Vallance BA, Finlay BB. Salmonella enterica serovar Typhimurium pathogenicity island 2 is necessary for complete virulence in a mouse model of infectious enterocolitis. Infect Immun 2005; 73:3219-27.

11. Chokr D, Cornu M, Neut C, Bortolus C, Charlet R, Desreumaux P, et al. Adherent invasive Escherichia coli (AIEC) strain LF82, but not Candida albicans, plays a profibrogenic role in the intestine. Gut Pathog 2021; 13:5.

12. Gordon IO, Bettenworth D, Bokemeyer A, Srivastava A, Rosty C, de Hertogh G, et al. Histopathology Scoring Systems of Stenosis Associated With Small Bowel Crohn's Disease: A Systematic Review. Gastroenterology 2020; 158:137-50 e1.

13. Salvador P, Macias-Ceja DC, Gisbert-Ferrandiz L, Hernandez C, Bernardo D, Alos R, et al. CD16+ Macrophages Mediate Fibrosis in Inflammatory Bowel Disease. J Crohns Colitis 2018; 12:589-99.

14. Ferrer-Font L, Mehta P, Harmos P, Schmidt AJ, Chappell S, Price KM, et al. High-dimensional analysis of intestinal immune cells during helminth infection. Elife 2020; 9.

15. Volk V, Reppas AI, Robert PA, Spineli LM, Sundarasetty BS, Theobald SJ, et al. Multidimensional Analysis Integrating Human T-Cell Signatures in Lymphatic Tissues with Sex of Humanized Mice for Prediction of Responses after Dendritic Cell Immunization. Front Immunol 2017; 8:1709.

16. Ying W, Riopel M, Bandyopadhyay G, Dong Y, Birmingham A, Seo JB, et al. Adipose Tissue Macrophage-Derived Exosomal miRNAs Can Modulate In Vivo and In Vitro Insulin Sensitivity. Cell 2017; 171:372-84 e12.

| Table S1. Characteristics of AIEC strains isolated from CD terminal ileum | | | | |
| --- | --- | --- | --- | --- |
| Strain | ^a^Adhesion index (ADH_I) | ^b^Invasion index (INV_I) | ^c^Replication index (REPL_I) % | Phenotype |
| CD2a | 2.38±1.75 | 0.13±0.06 | 414.73±178.14 | AIEC |
| CD2b | 2.31±0.77 | 0.23±0.06 | 172.68±113.49 | AIEC |
| CD2c | 1.80±0.62 | 3.58±0.95 | 73.06±20.60 | non-AIEC |
| CD2d | 3.40±0.49 | 7.25±2.65 | 116.50±50.92 | AIEC |
| CD3a | 2.10±0.12 | 0.22±0.11 | 69.47±37.39 | non-AIEC |
| CD3c | 0.05±0.02 | 0.12±0.06 | 56.32±22.75 | non-AIEC |
| CD4a | 2.44±0.25 | 2.15±0.10 | 290.81±75.37 | AIEC |
| CD4c | 3.60±0.67 | 1.06±0.24 | 88.64±24.17 | non-AIEC |
| CD5a | 0.08±0.01 | 0.06±0.02 | 78.69±32.09 | non-AIEC |
| CD6b | 0.41±0.06 | 0.24±0.08 | 88.08±28.50 | non-AIEC |
| CD7a | 1.69±0.35 | 1.62±1.19 | 318.22±163.96 | AIEC |
| CD7b | 4.80±0.35 | 1.53±0.64 | 226.84±30.71 | AIEC |
| CD8a | 5.13±1.11 | 0.27±0.21 | 510.74±188.60 | AIEC |
| CD8c | 3.62±1.62 | 0.23±0.11 | 451.05±177.00 | AIEC |
| CD9b | 2.80±1.12 | 6.07±2.80 | 35.98±12.35 | non-AIEC |
| CD9c | 0.13±0.04 | 0.17±0.11 | 53.48±38.76 | non-AIEC |
| CD10a | 0.07±0.04 | 0.10±0.03 | 48.17±16.65 | non-AIEC |
| CD11a | 1.50±0.84 | 0.35±0.11 | 177.59±72.08 | AIEC |
| CD11b | 2.72±0.75 | 0.27±0.08 | 309.70±110.54 | AIEC |
| CD11c | 2.25±0.42 | 0.36±0.12 | 271.90±104.34 | AIEC |
| CD12b | 0.07±0.02 | 0.07±0.02 | 34.29±2.70 | non-AIEC |
| CD13a | 0.08±0.02 | 0.09±0.03 | 71.92±28.31 | non-AIEC |
| CD13b | 0.59±0.23 | 0.18±0.05 | 92.88±37.60 | non-AIEC |
| CD15a | 2.67±1.12 | 0.19±0.06 | 313.00±27.04 | AIEC |
| CD15b | 2.39±1.31 | 0.35±0.13 | 281.71±293.10 | AIEC |
| CD15c | 1.96±0.36 | 0.95±0.56 | 203.48±81.42 | AIEC |
| CD16a | 1.17±0.14 | 0.33±0.05 | 84.43±12.91 | non-AIEC |
| CD17a | 1.82±0.55 | 2.20±0.96 | 178.84±74.18 | AIEC |
| CD17c | 2.66±1.12 | 0.80±0.26 | 224.24±188.12 | AIEC |
| CD17d | 0.57±0.26 | 0.28±0.06 | 26.75±15.78 | non-AIEC |
| CD19a | 1.31±0.33 | 2.18±0.93 | 165.78±88.33 | AIEC |
| CD19c | 3.54±0.22 | 0.42±0.15 | 149.48±102.24 | AIEC |
| CD19d | 1.18±0.58 | 0.20±0.13 | 65.04±29.78 | non-AIEC |
| CD20a | 0.33±0.15 | 0.11±0.04 | 113.75±69.67 | non-AIEC |
| CD20b | 0.09±0.01 | 0.43±0.32 | 92.20±77.15 | non-AIEC |
| CD20c | 0.08±0.02 | 1.01±0.30 | 60.27±17.71 | non-AIEC |
| CD21a | 0.66±0.17 | 1.70±1.10 | 49.31±12.95 | non-AIEC |
| CD21b | 1.18±0.63 | 0.11±0.05 | 54.11±7.03 | non-AIEC |
| LF82 | 2.70±0.64 | 6.73±1.87 | 307.95±107.70 | AIEC reference strain |
| K12 | 0.73±0.40 | 0.04±0.01 | 64.27±54.77 | Nonpathogenic *E. coli* strain |
| ^a^Mean number of bacteria per Caco-2 cell after 3h of incubation.  ^b^Mean ratio of the original inoculum after 1h gentamicin treatment of infected Caco-2 cells.  ^c^Percentage of intracellular bacteria at 24 h post infection relative to the number after 1h gentamicin treatment, defined as 100%.  Data represent mean and standard errors of three independent experiments made in duplicate. | | | | |

| Table S2. Primer sequences used in qPCR | | | |
| --- | --- | --- | --- |
| Gene | Species | Forward sequence | Reverse sequence |
| TNF-α | mouse | CGCTGAGGTCAATCTGC | GGCTGGGTAGAGAATGGA |
| IFN-γ | mouse | CAGCAACAGCAAGGCGAAA | CTGGACCTGTGGGTTGTTGAC |
| IL-1β | mouse | AGTTGACGGACCCCAAA | TCTTGTTGATGTGCTGCTG |
| TGFβ1 | mouse | TCTGCATTGCACTTATGCTGA | AAAGGGCGATCTAGTGATGGA |
| Col1a1 | mouse | CAGGCTGGTGTGATGGGATT | CAGGCTGGTGTGATGGGATT |
| α-SMA | mouse | GTCCCAGACATCAGGGAGTAA | TCGGATACTTCAGCGTCAGGA |
| Fibronectin | mouse | CGAAGCCGGGAAGAGCAAG | CGTTCCCACTGCTGATTTATCTG |
| CD16 | mouse | CAGAATGCACACTCTGGAAGC | GGGTCCCTTCGCACATCAG |
| MMP3 | mouse | CGGTGGCTTCAGTACCTTTC | ACCTCCTCCCAGACCTTCA |
| TIMP1 | mouse | CAGATACCATGATGGCCCCC | TCTGGTAGTCCTCAGAGCCC |
| 36B4 | mouse | AGATGCAGCAGATCCGCAT | GTTCTTGCCCATCAGCACC |
| let-7b | mouse | CGGGCTGAGGTAGTAGGTTG | CAGCCACAAAAGAGCACAAT |
| TGFβ1 | human | TGGGGACTTCTTCTTGGCACT | ATAGGGGCGTCTGAGGAAC |
| COL1A1 | human | CTGCTGGACGTCCTGGTGAA | ACGCTGTCCAGCAATACCTTGA |
| α-SMA | human | CGTGGCTACTCCTTCGTG | TGATGACCTGCCCGTCT |
| Fibronectin | human | CTATTTA CCAACCCCAGACCC | GCATTCCCACAGAGTAGACCA |
| GAPDH | human | GGATGCTGCCCTTACCC | GTTCACACCGACCTTCACC |
| let-7b | human | GGGGTGAGGTAGTAGATTGT | TGAGGTGCTGTGCGTGAC |
| U6 | mouse/human | ATTGGAACGATACAGAGAAGATT | GGAACGCTTCACGAATTTG |

**Figure** **legends:**

Figure S1.

(A) Macroscopic appearance and lengths of colons in different groups. (B) The mRNA levels of TNF-α, IFN-γ and IL-1β in the colon tissue determined by qPCR. Data were normalized to mouse 36B4 RNA. (C) Protein levels of TGFβ1, Col1a1, α-SMA and fibronectin in the colonic tissue of WT, IL-10^-/-^ and IL-10^-/-^ mice with AIEC or K12 infection. The densitometric intensity of the bands was quantified using ImageJ software. Data are expressed as the means ± SEM, n=5 in each group. *P<0.05, **P<0.01, ***P<0.001.

Figure S2.

(A&B) Macroscopic observation and lengths of colons in different groups. (C) Protein levels of TGFβ1, Col1a1, α-SMA and fibronectin in the colonic tissue in different groups. The densitometric intensity of the bands was quantified using ImageJ software. Data are expressed as the means ± SEM, n=5 in each group. *P<0.05, **P<0.01, ***P<0.001.

Figure S3.

(A) Immunofluorescence staining for the human intestinal Mø marker CD68 and FISH for let-7b were performed on terminal ileum specimens of AIEC(-) and AIEC(+) CD patients. (Red, let-7b; Green, CD68; Blue, DPAI). (B) The miRNA levels of let-7b in the colonic lamina propria Møs from IL-10^-/-^ mice with/without AIEC infection. Data are expressed as the means ± SEM, n=5 in each group. **P<0.01. (C) Correlation between the proportion of CD206+CD16+ Mø and fibrosis score in the colonic tissue of IL-10^-/-^ mice with different treatments. Spearman’s test was used for correlation analysis. AIEC(-): AIEC-negative colonization; AIEC(+): AIEC-positive colonization.

Figure S4.

(A) Macroscopic observation and lengths of colons in different groups. (B) Protein levels of TGFβ1, Col1a1, α-SMA and fibronectin in the colonic tissue of IL-10^-/-^ after different treatments. The densitometric intensity of the bands was quantified using ImageJ software. Data are expressed as the means ± SEM, n=5 in each group. *P<0.05, **P<0.01, ***P<0.001.

Figure S5.

(A) Protein levels of TGFβ1, TIMP-1 and MMP3 of RAW264.7 cell with let-7b-agomir or -antagomir transfection. The densitometric intensity of the bands was quantified using ImageJ software. Data are expressed as the means ± SEM. All the experiments repeated 3 times independently.
