# Supplementary figures and images for: Crohn’s disease-associated AIEC inhibiting intestinal epithelial cell-derived exosomal let-7b expression regulates macrophage polarization to exacerbate intestinal fibrosis
Source: Gut Microbes. 2023 Mar 21;15(1):2193115. doi: 10.1080/19490976.2023.2193115 (PMC10038049; doi:10.1080/19490976.2023.2193115)

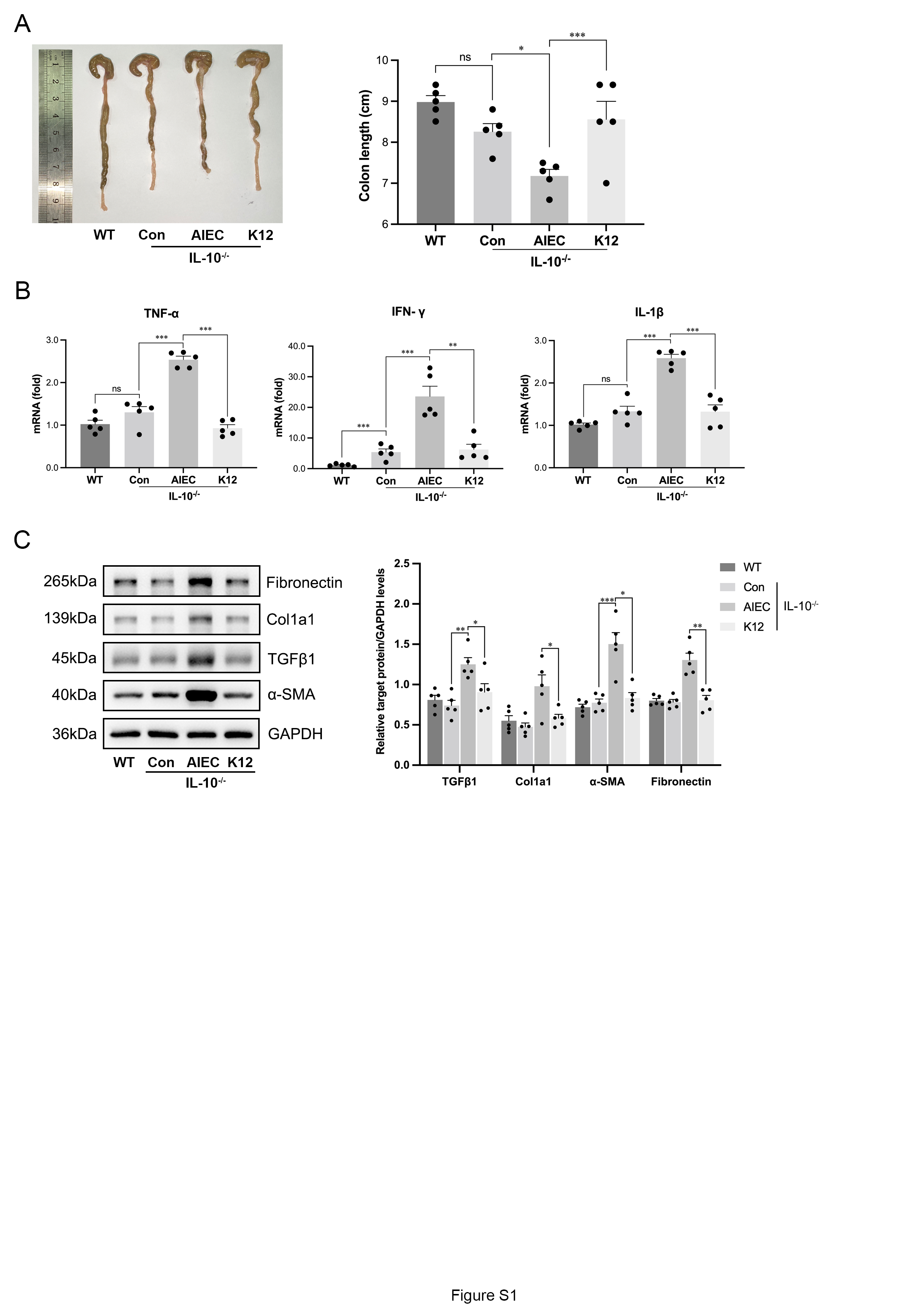

Supplement: Supplemental Material [file KGMI_A_2193115_SM6628.zip › FigS1_revision_publish.tif]

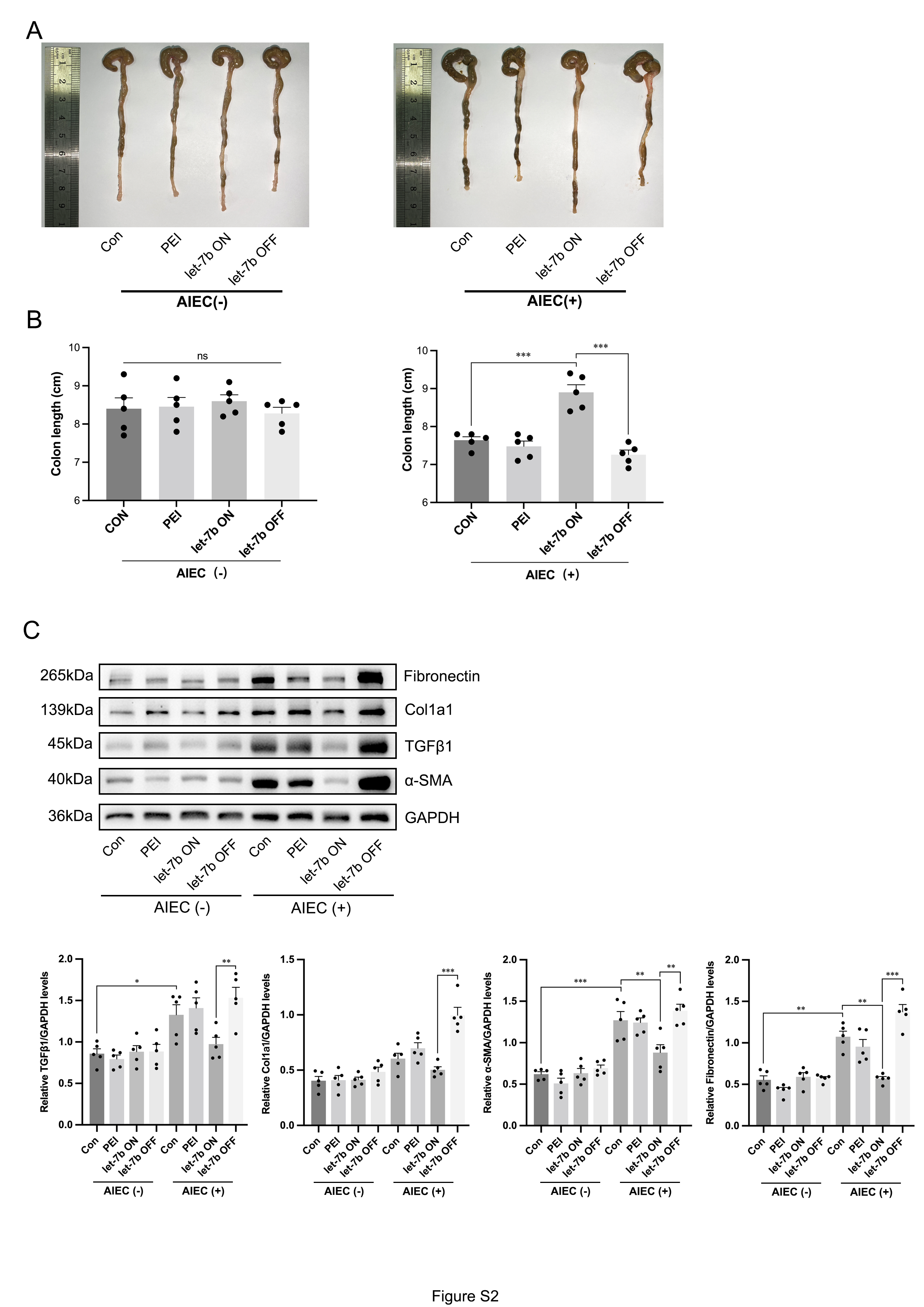

Supplement: Supplemental Material [file KGMI_A_2193115_SM6628.zip › FigS2_revision_publish.tif]

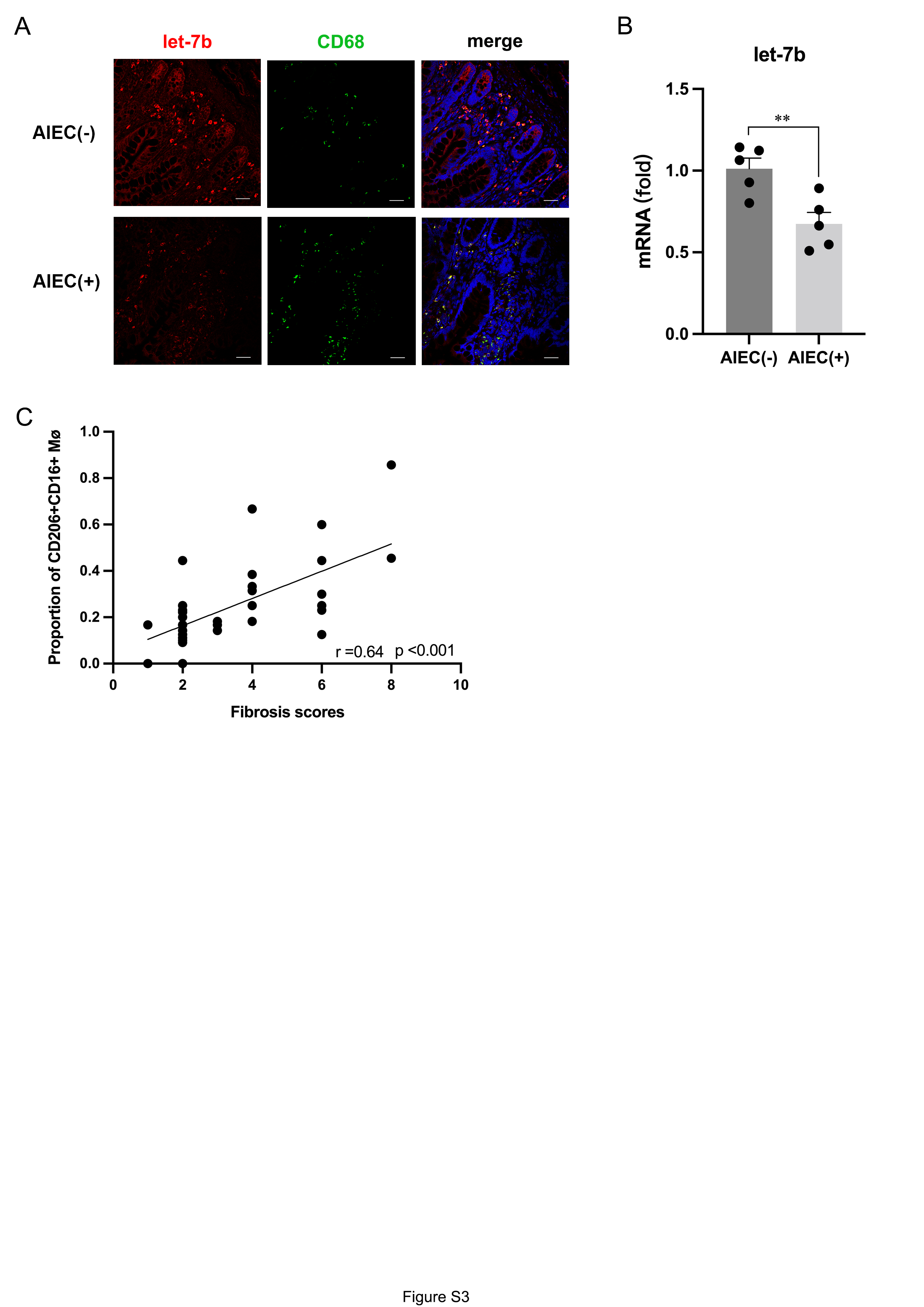

Supplement: Supplemental Material [file KGMI_A_2193115_SM6628.zip › FigS3_revision_publish.tif]

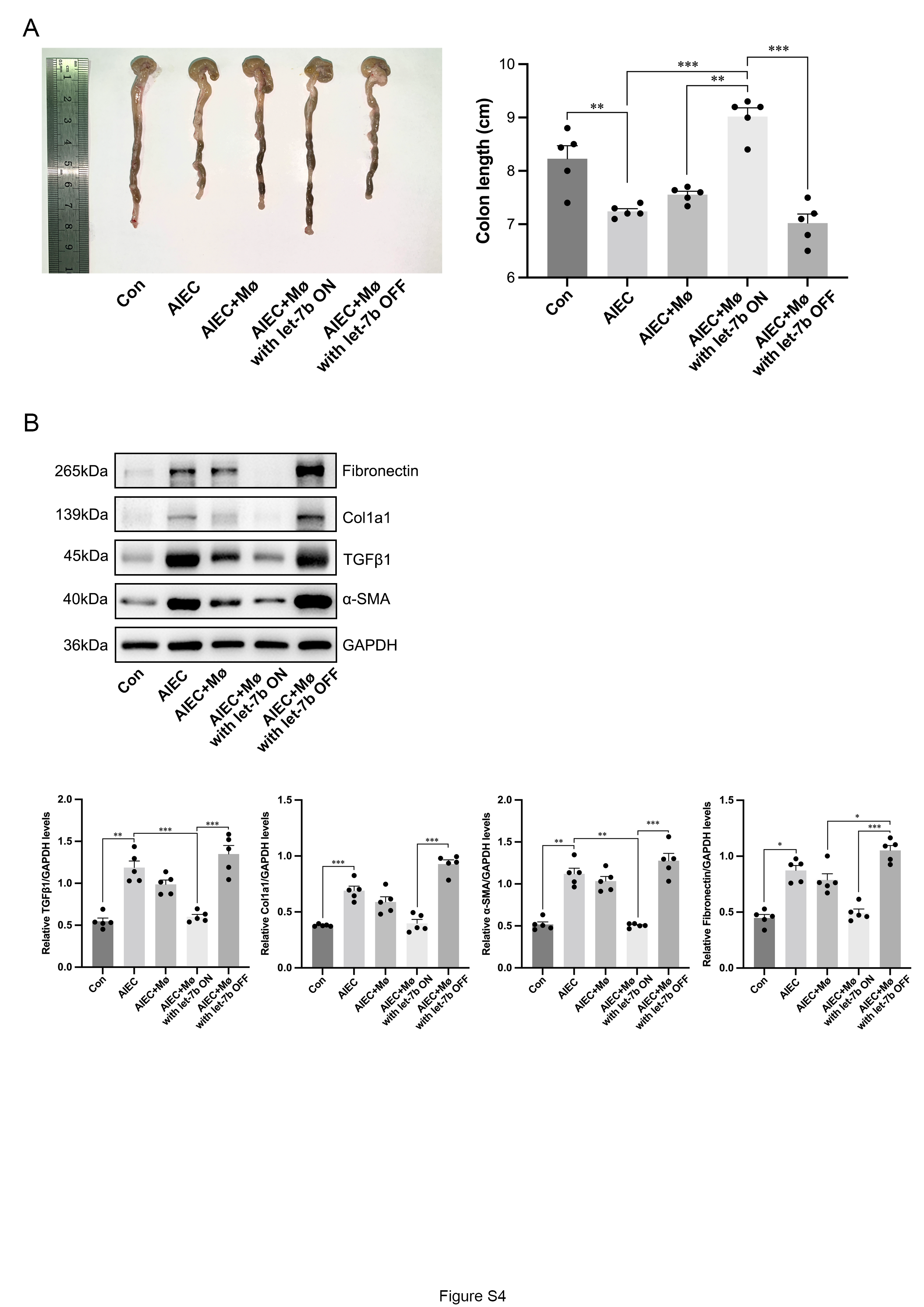

Supplement: Supplemental Material [file KGMI_A_2193115_SM6628.zip › FigS4_revision_publish.tif]

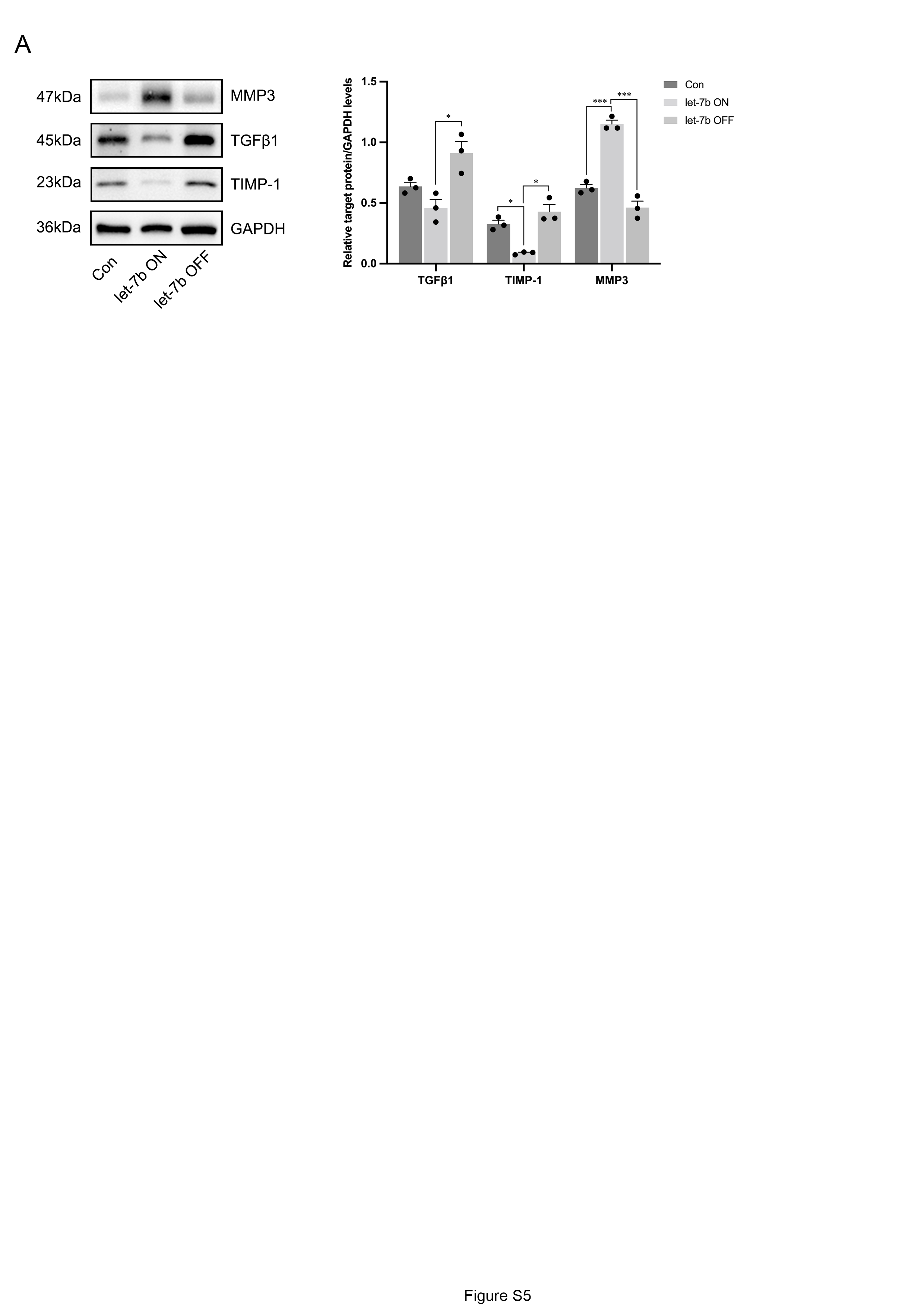

Supplement: Supplemental Material [file KGMI_A_2193115_SM6628.zip › FigS5_revision_publish.tif]
